# Supplementary material for: Upregulation of the Mevalonate Pathway through EWSR1-FLI1/EGR2 Regulatory Axis Confers Ewing Cells Exquisite Sensitivity to Statins
Source: Cancers (Basel). 2022 May 8;14(9):2327. doi: 10.3390/cancers14092327 (PMC9100622; doi:10.3390/cancers14092327)
Supplement: Supplementary file 1 [file cancers-14-02327-s001.zip › File S1.pdf]

figure 1A

Chemidoc acquisition

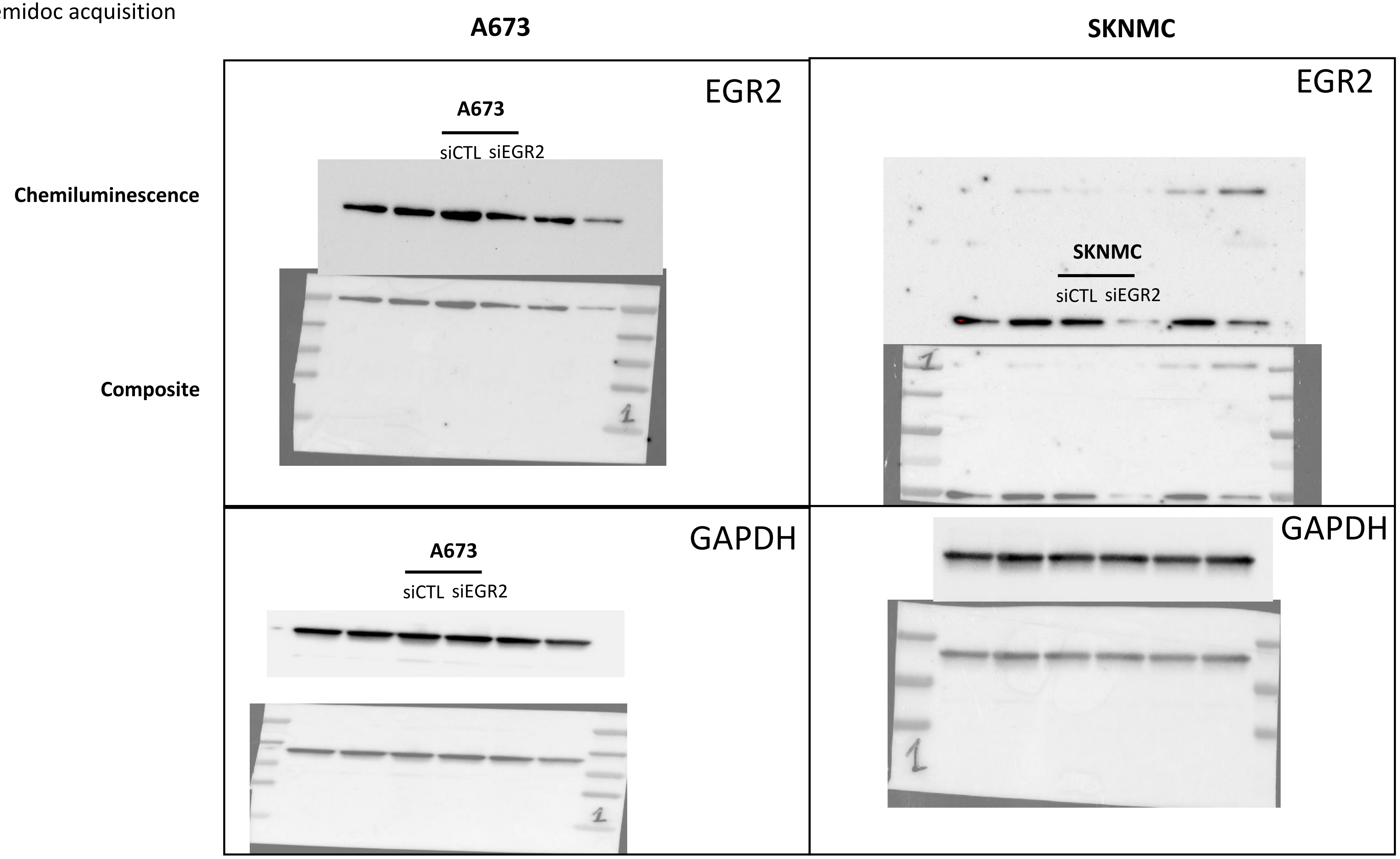

A673/TR/EF1 figure 2A

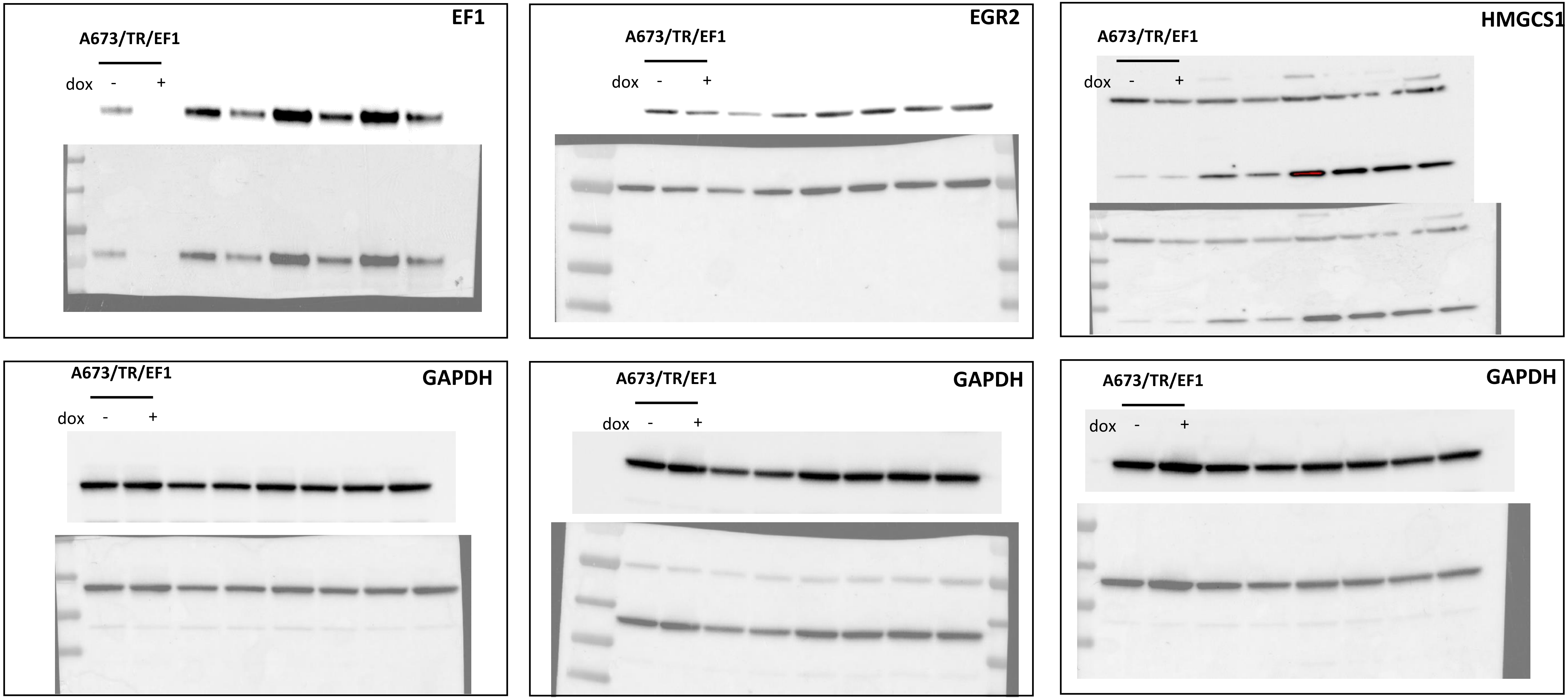

A673/TR/EF1 figure 2A

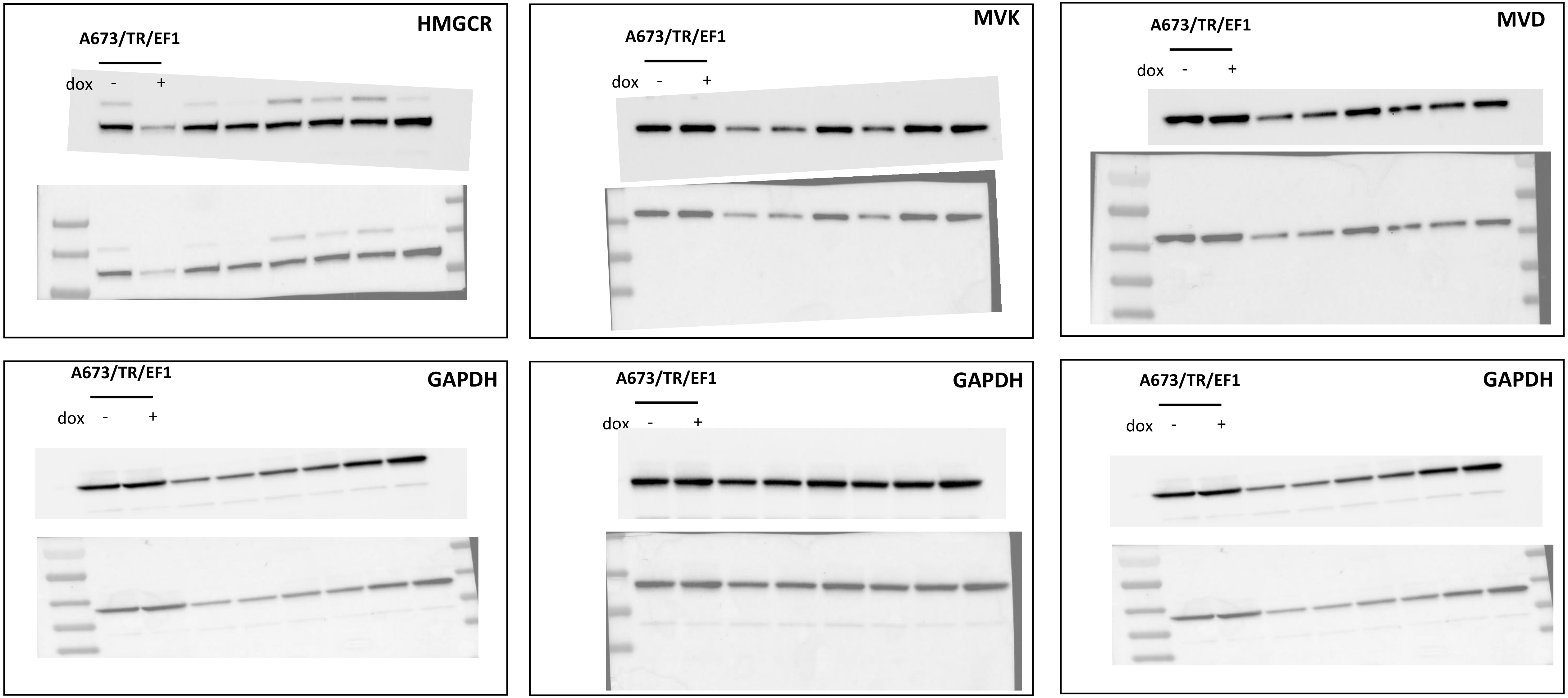

A673_siEF1 figure 2A

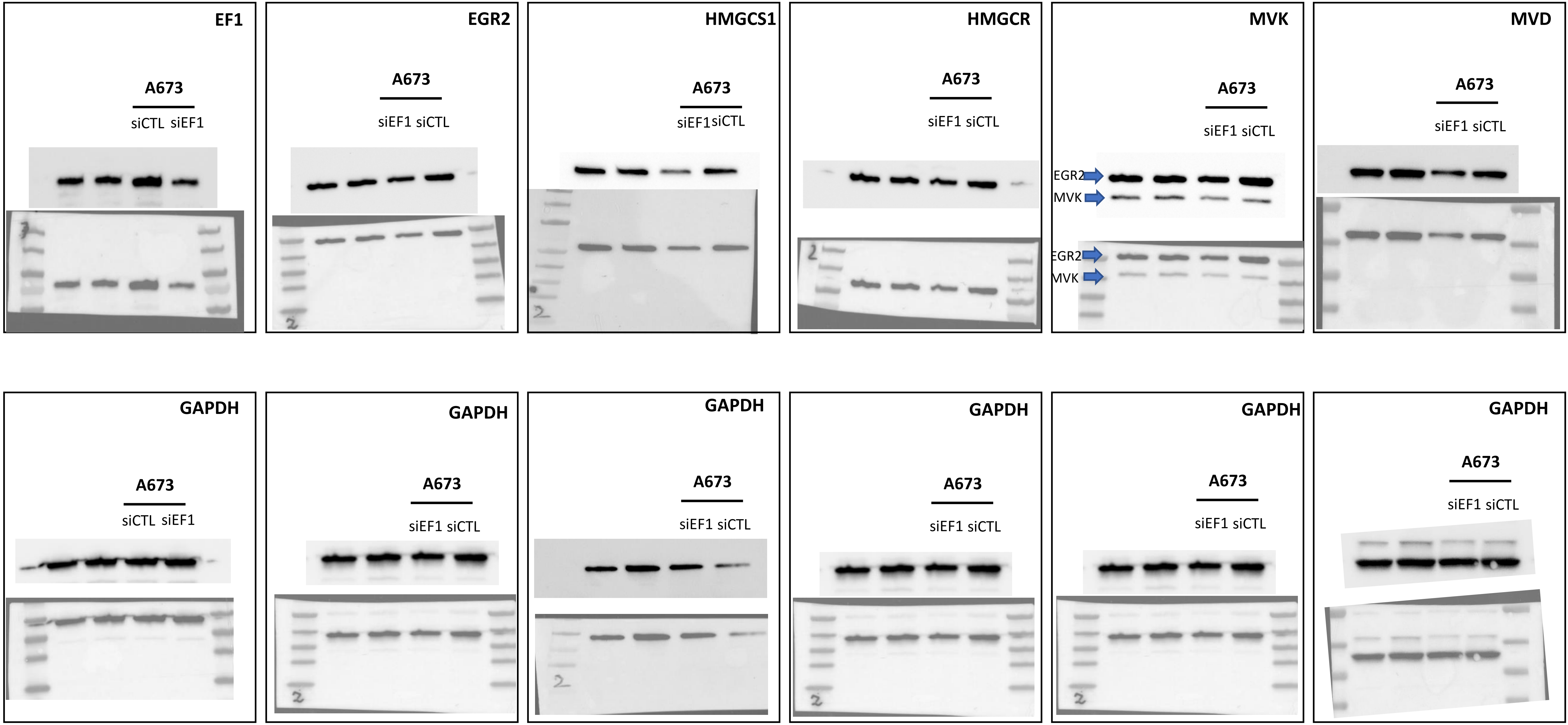

SKNMC_siEF1 figure 2A

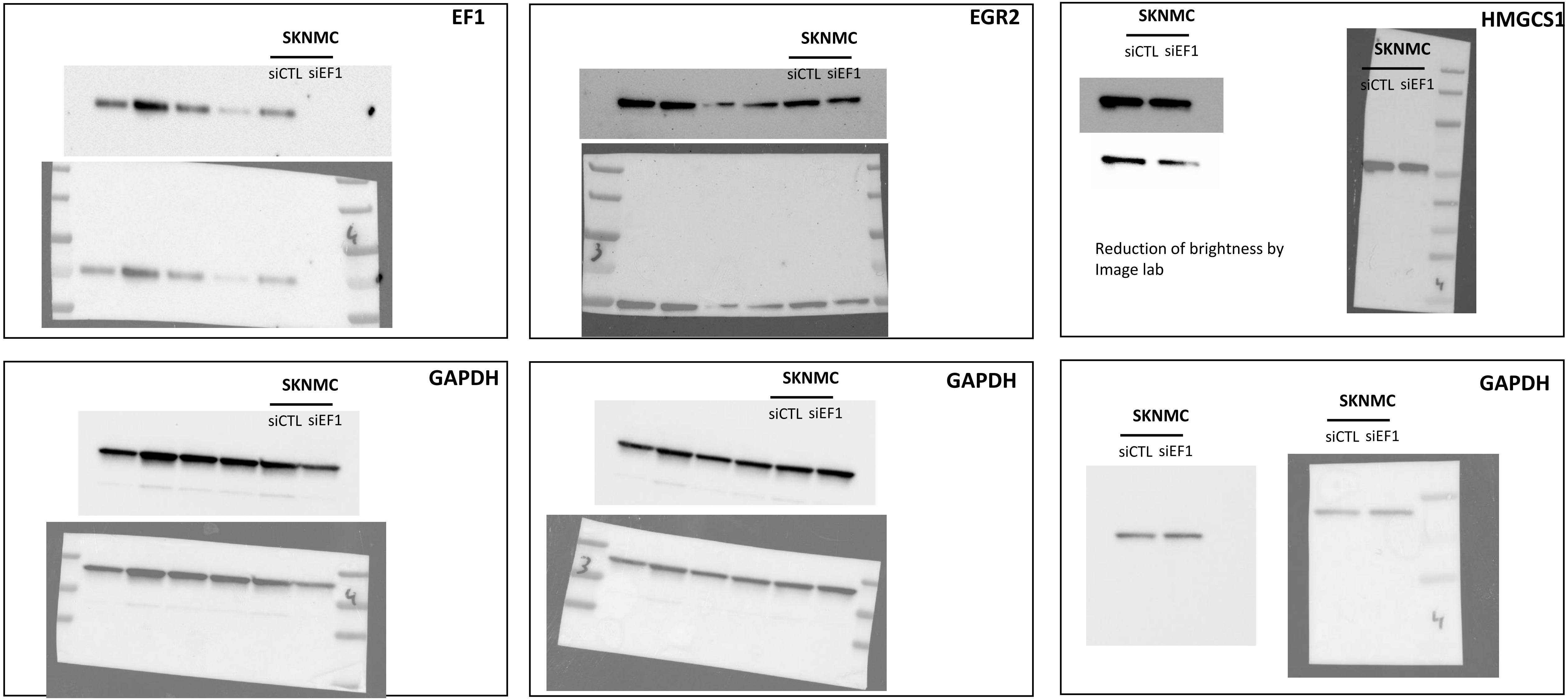

SKNMC_siEF1 figure 2A

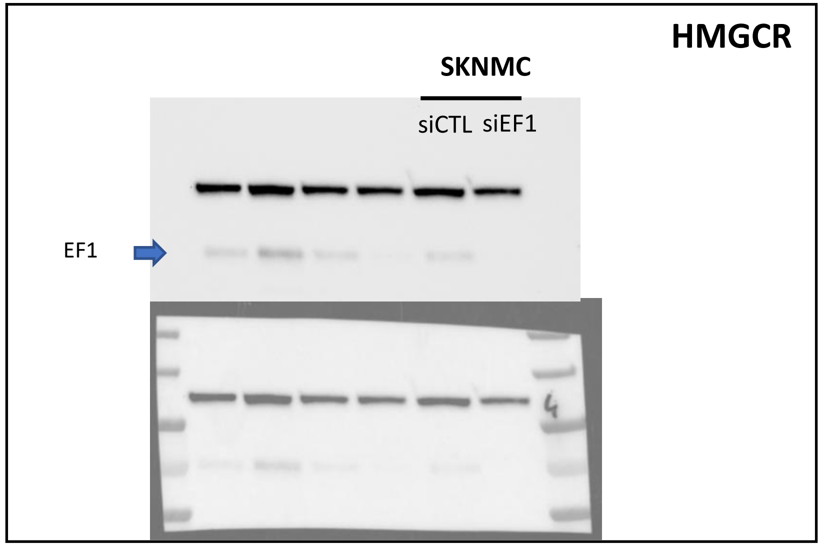

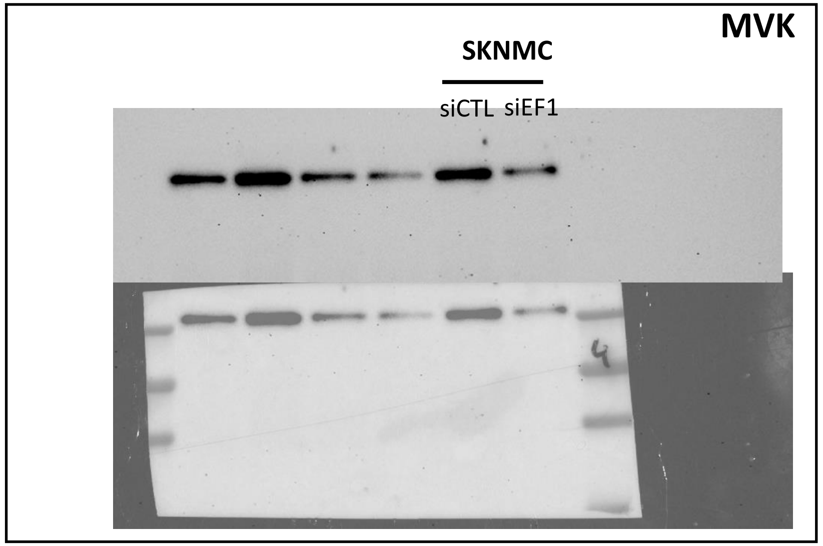

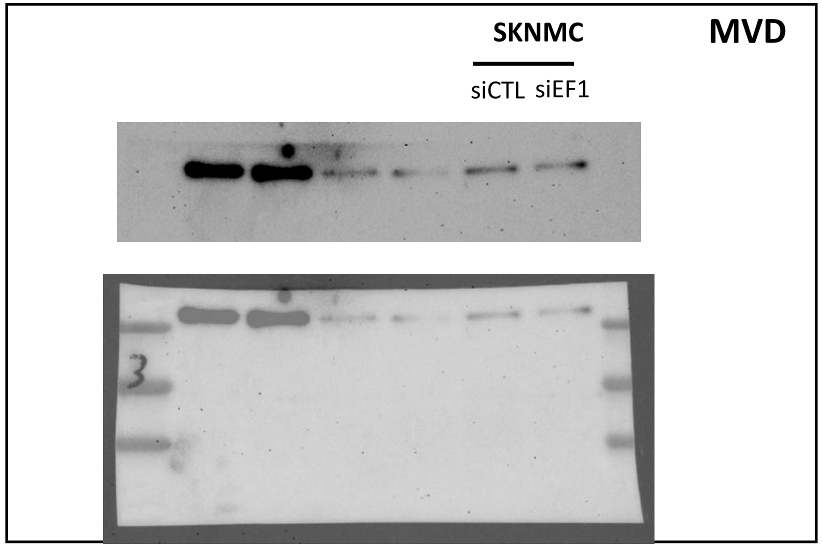

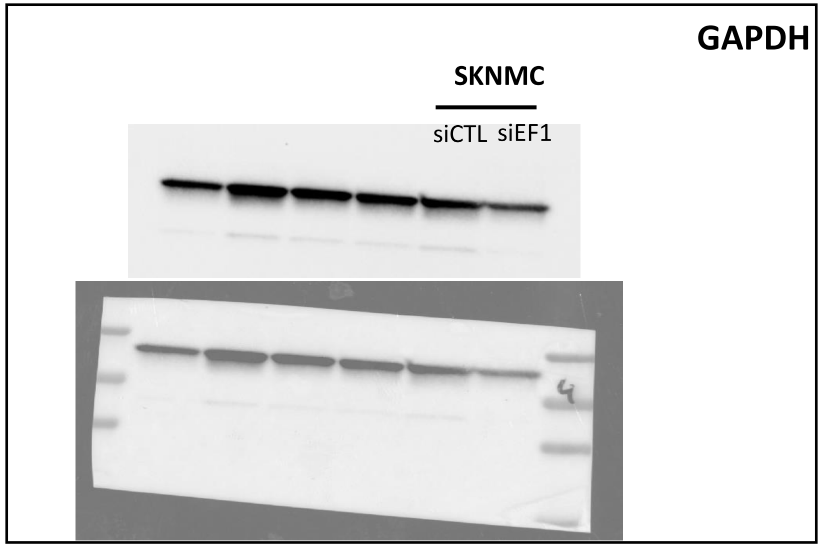

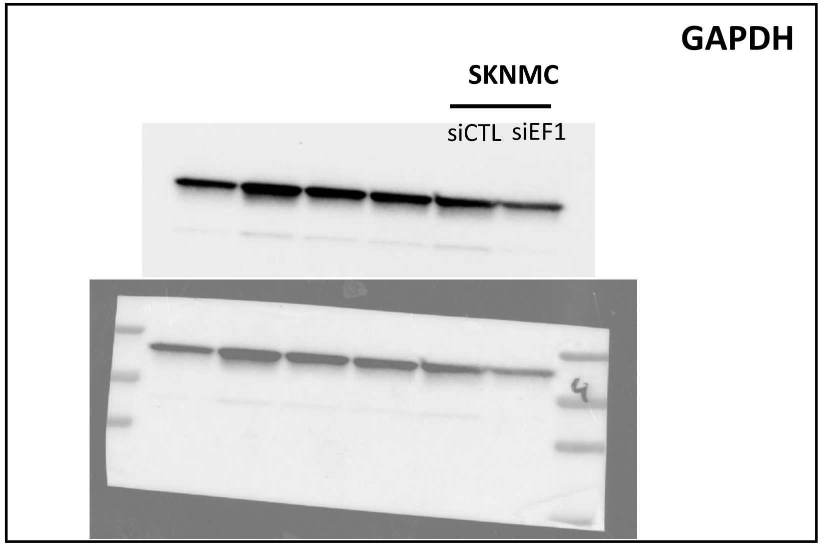

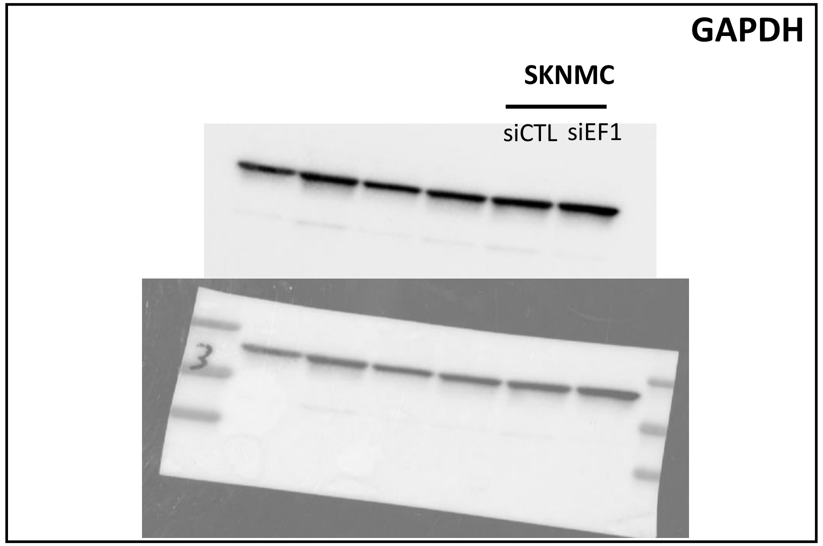

TC71_siEF1 figure 2A

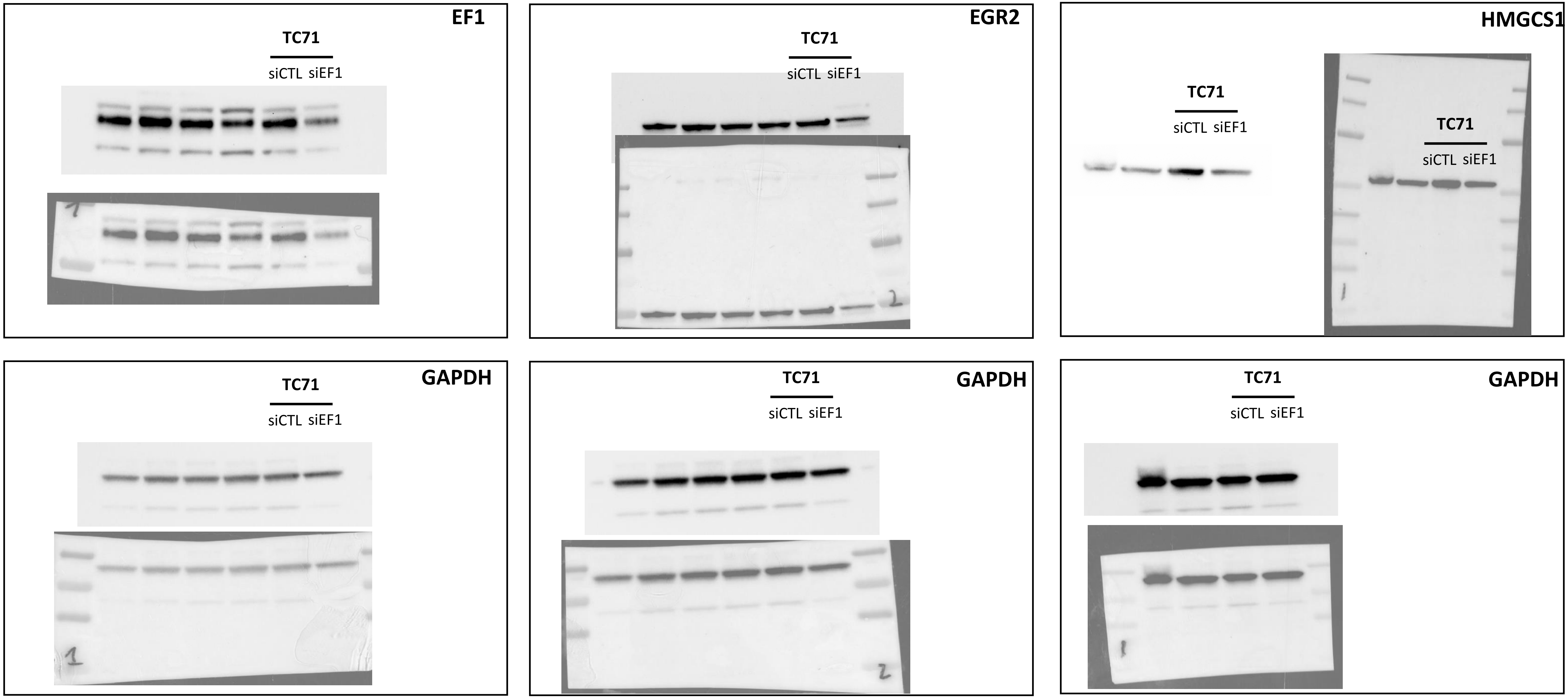

TC71_siEF1 figure 2A

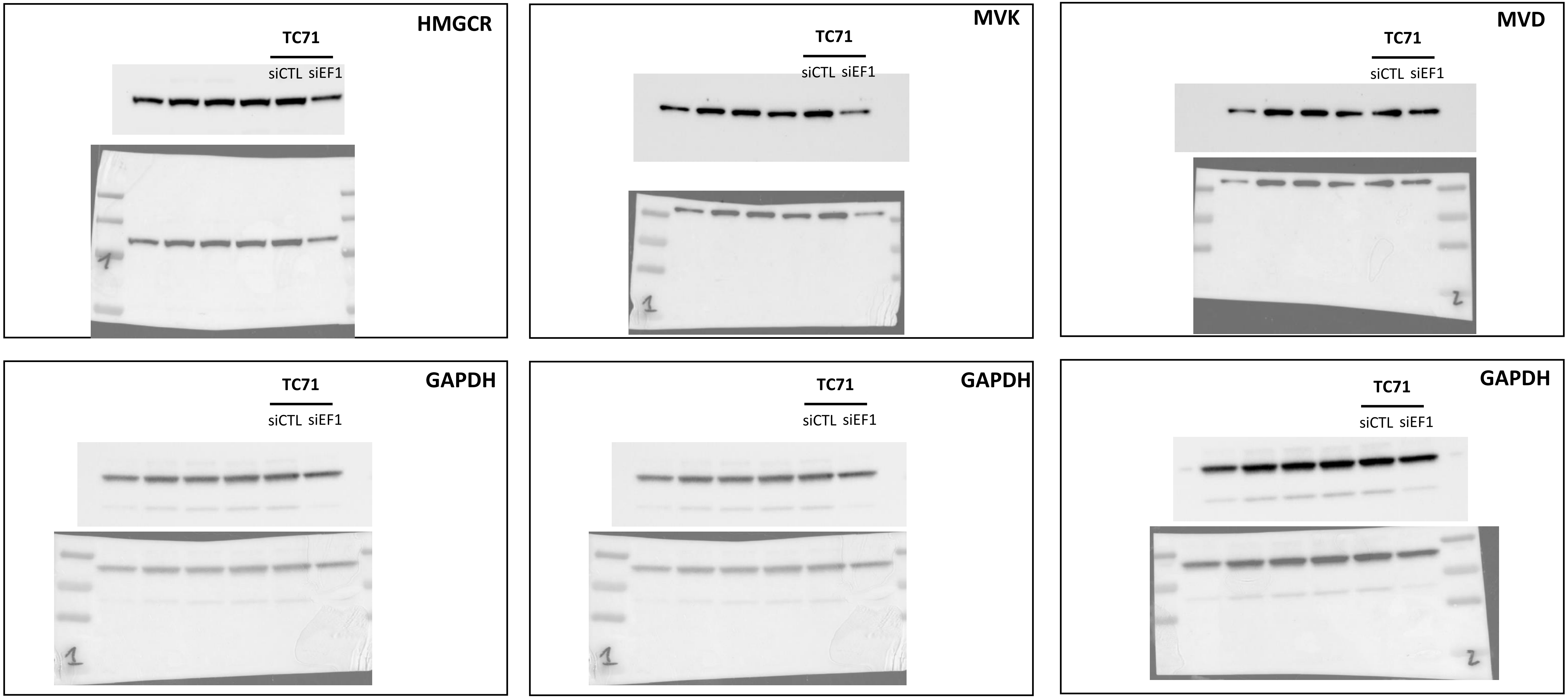

POE_siEF1 figure 2A

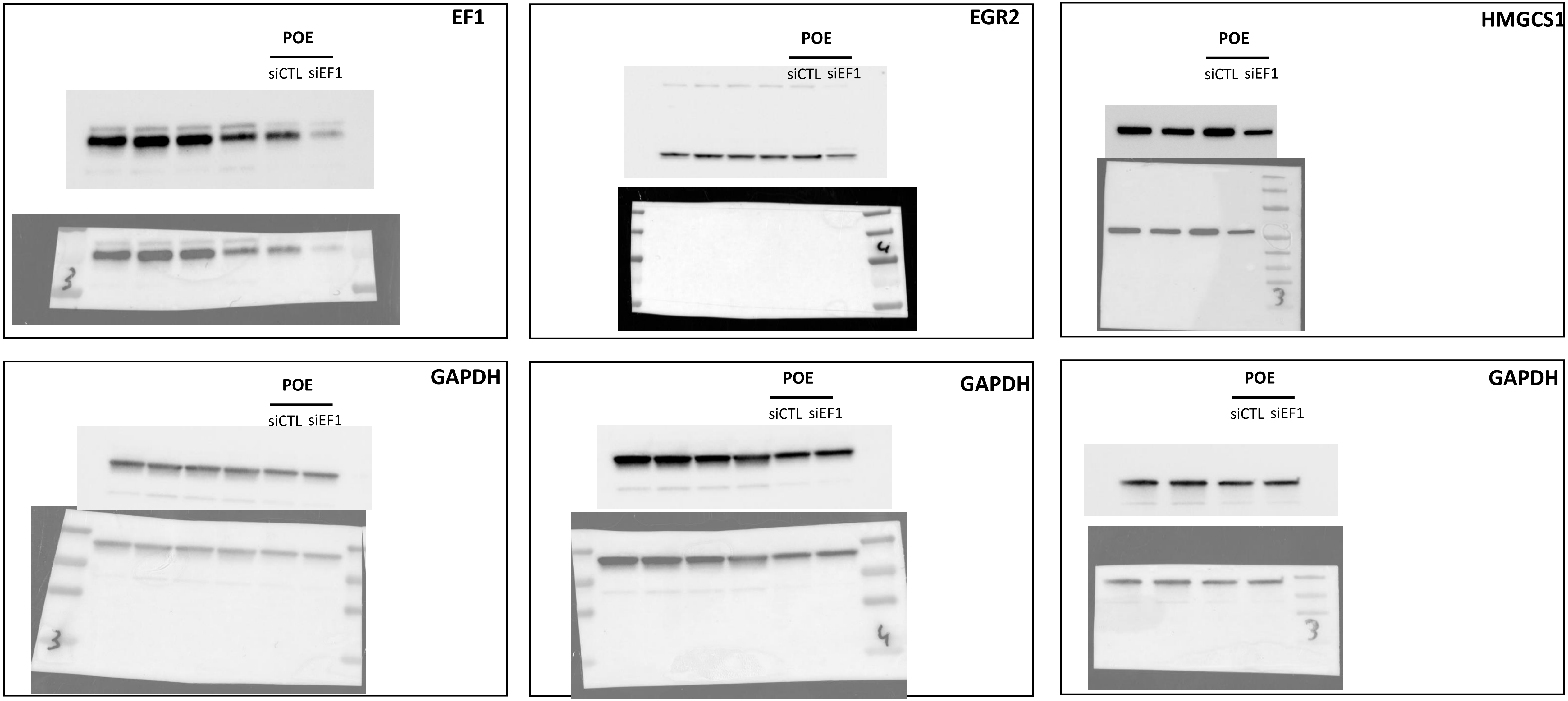

POE_siEF1 figure 2A

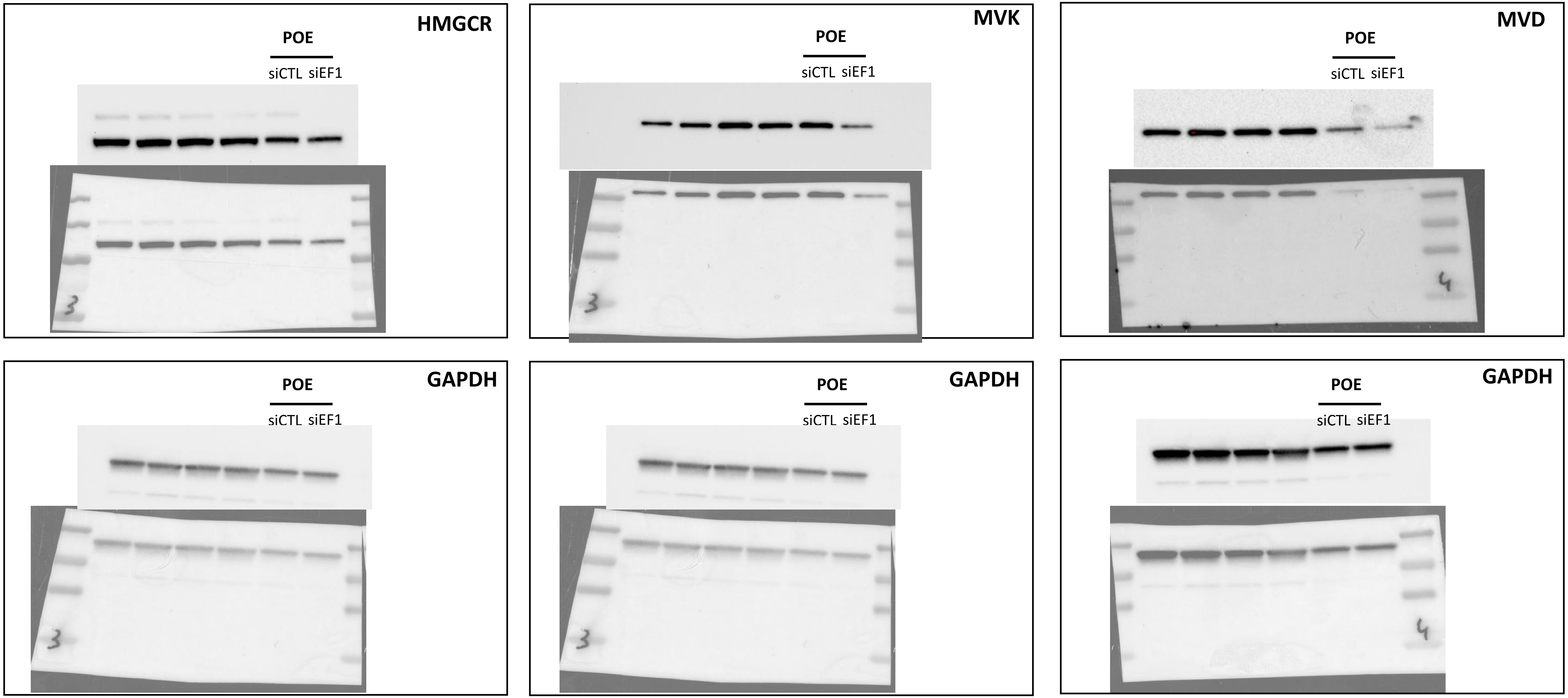

A673_siEGR2 figure 2B

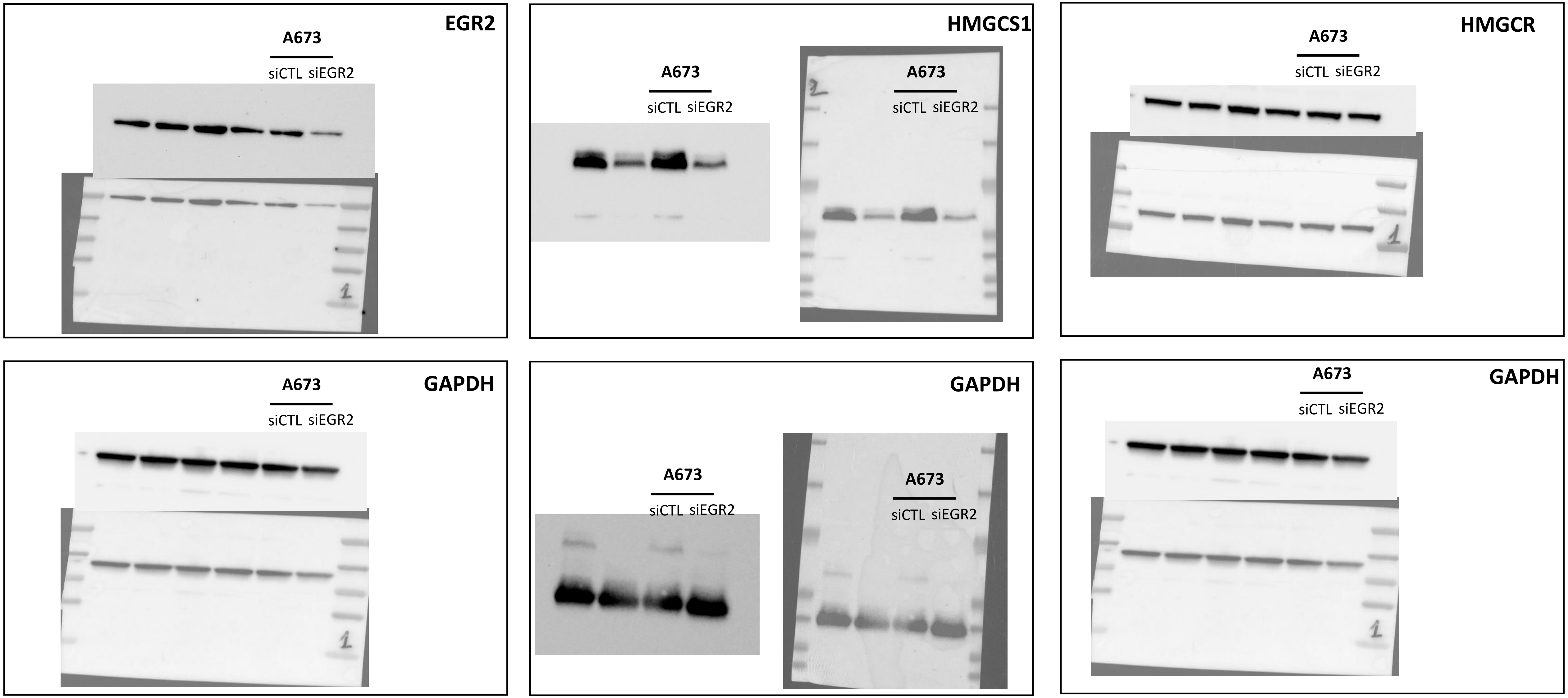

A673_siEGR2 figure 2B

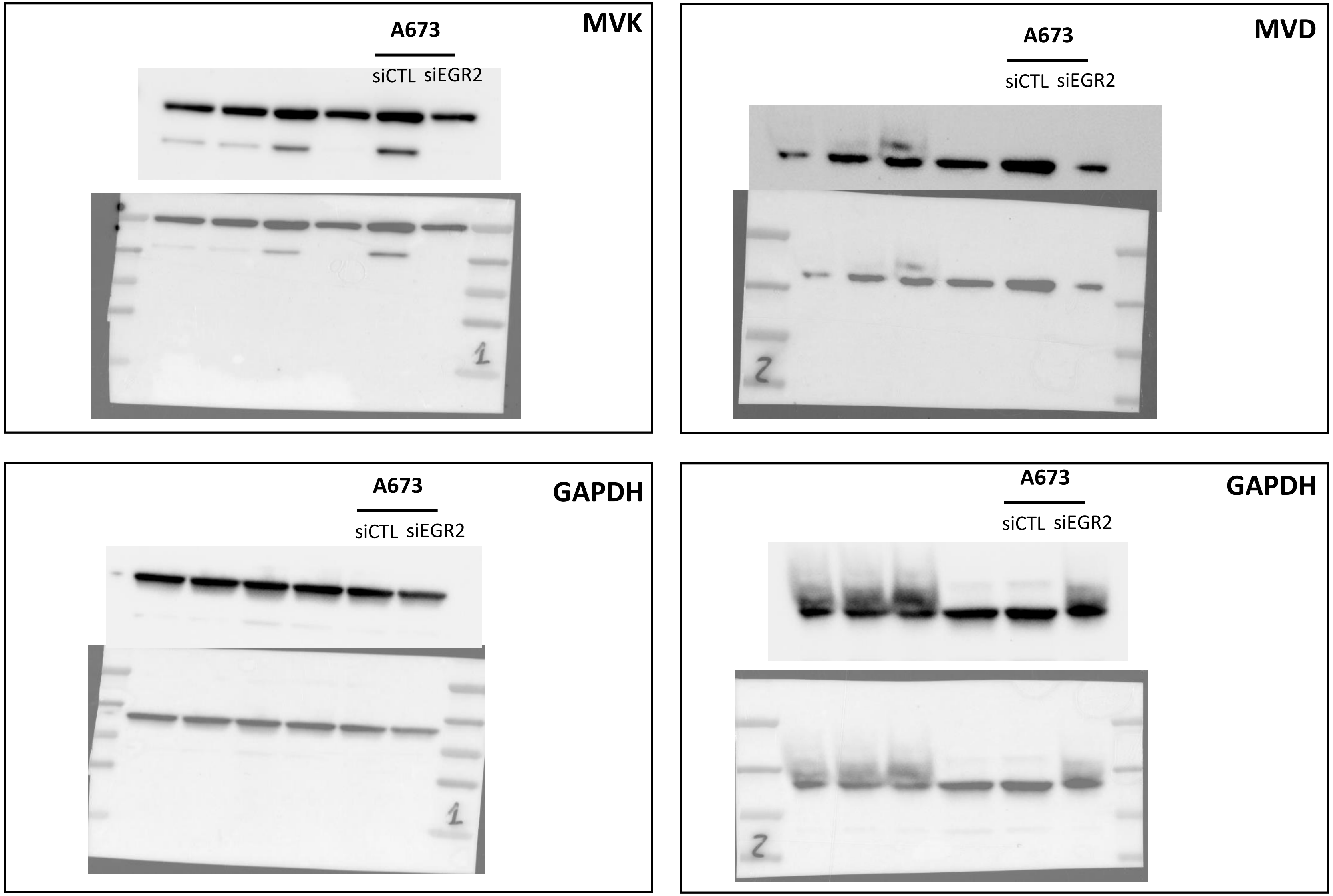

SKNMC_siEGR2 figure 2B

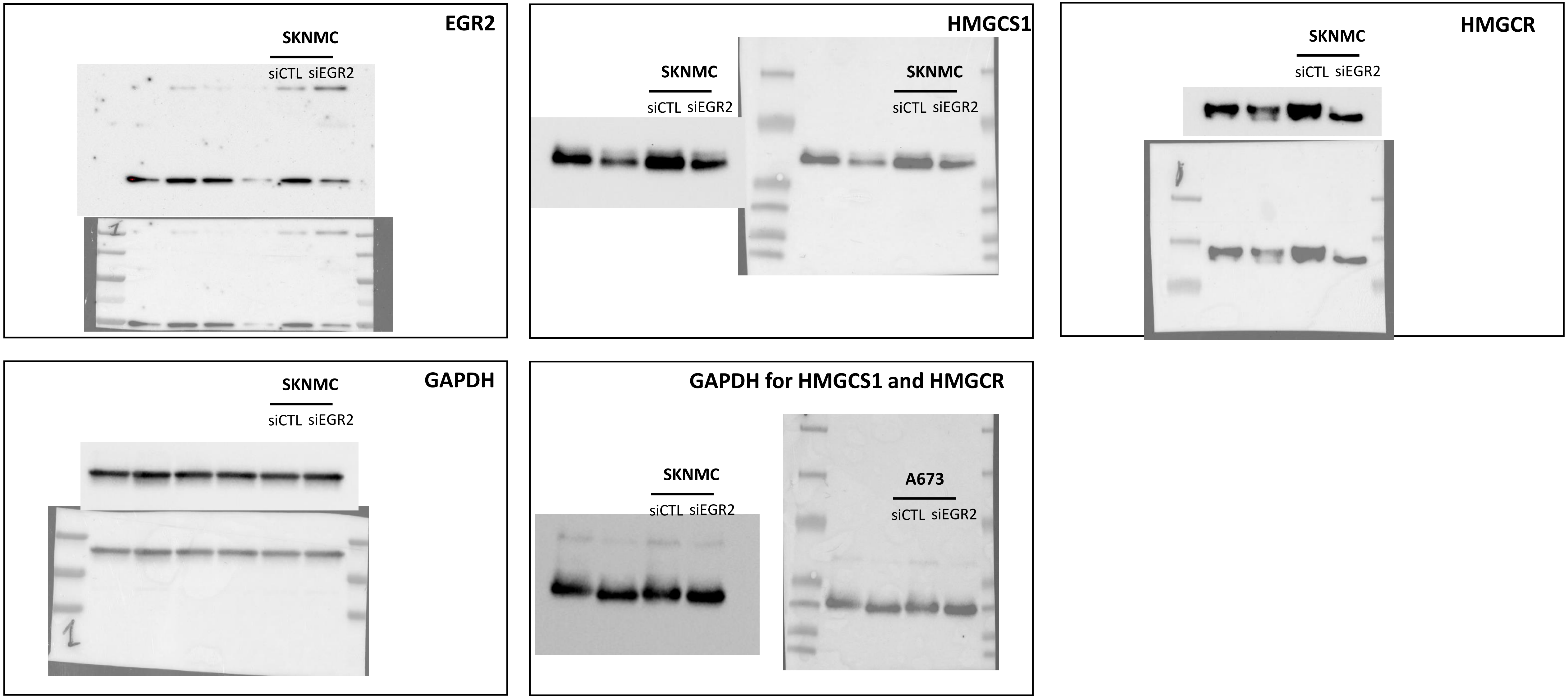

SKNMC_siEGR2 figure 2B

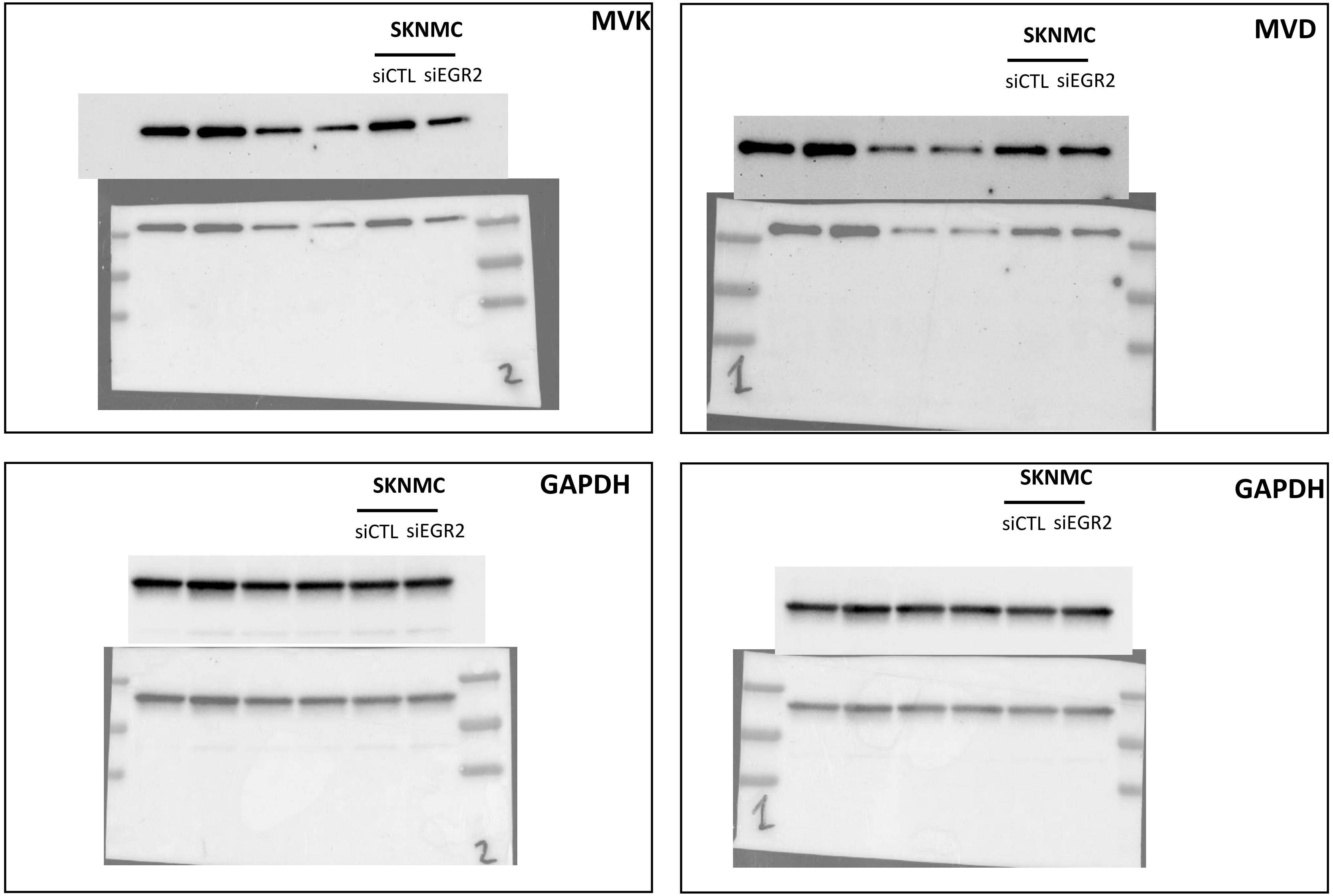

TC71_siEGR2 figure 2B

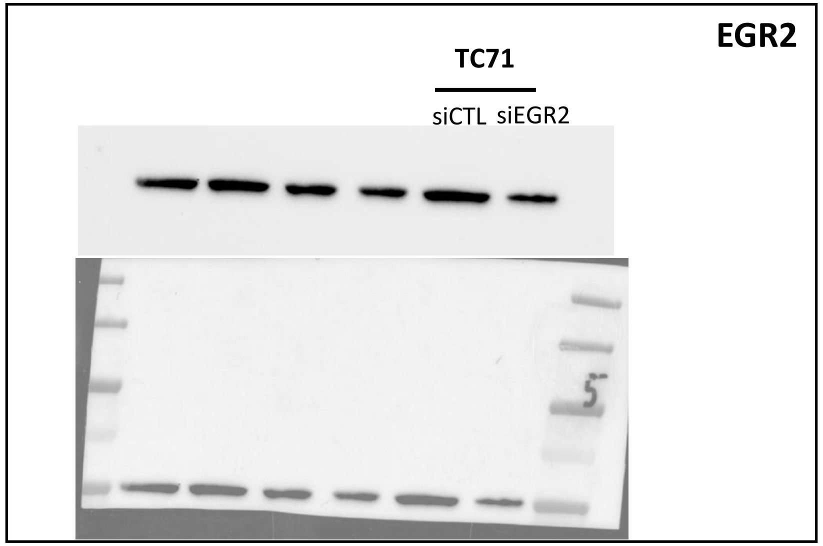

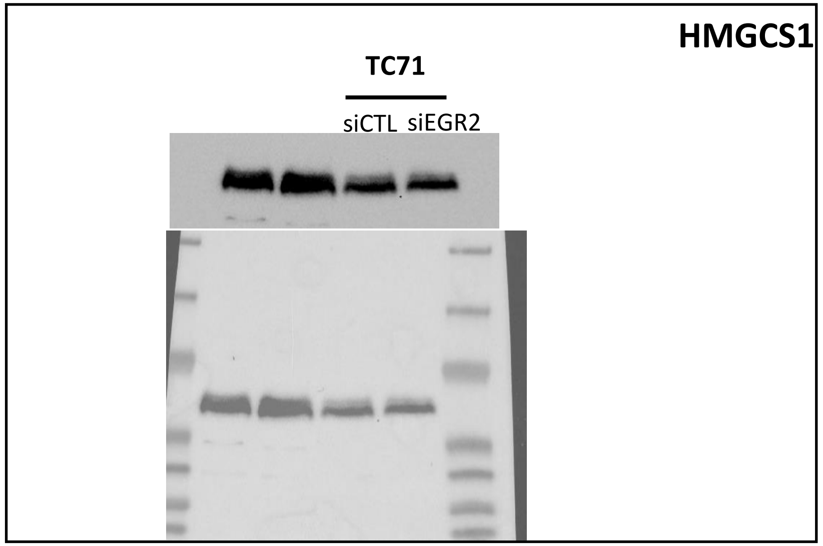

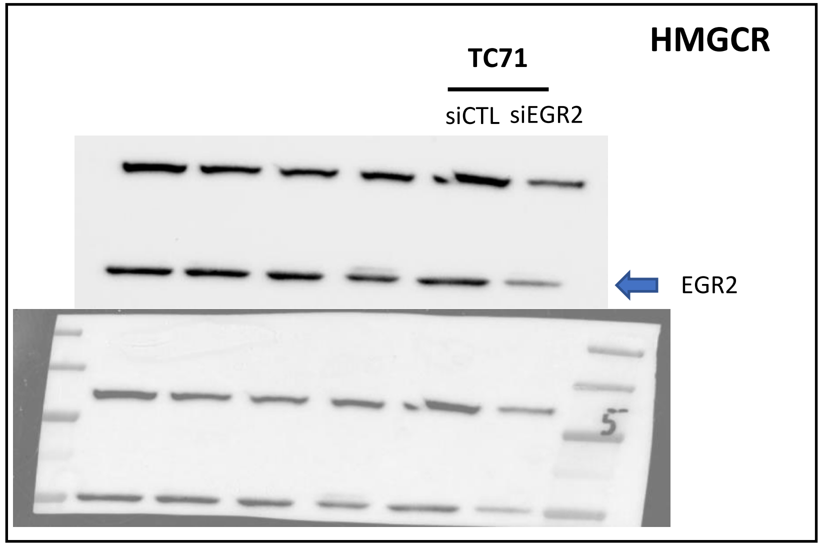

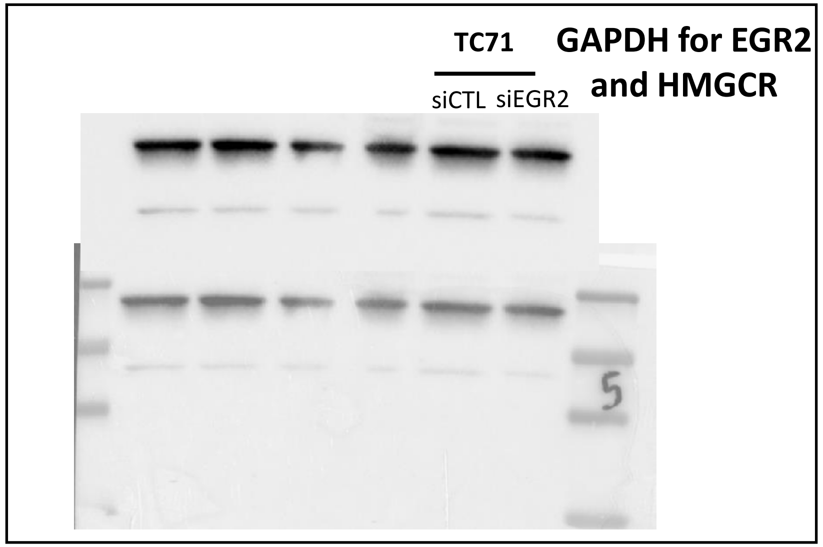

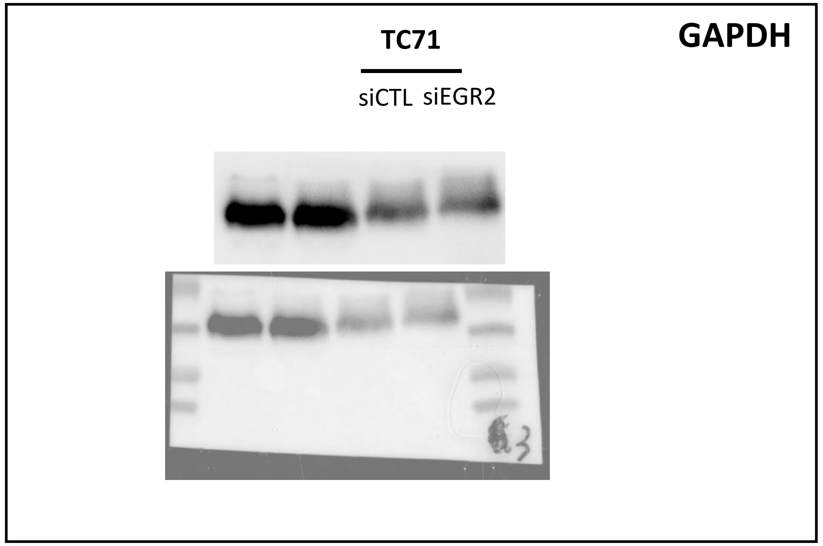

TC71_siEGR2 figure 2B

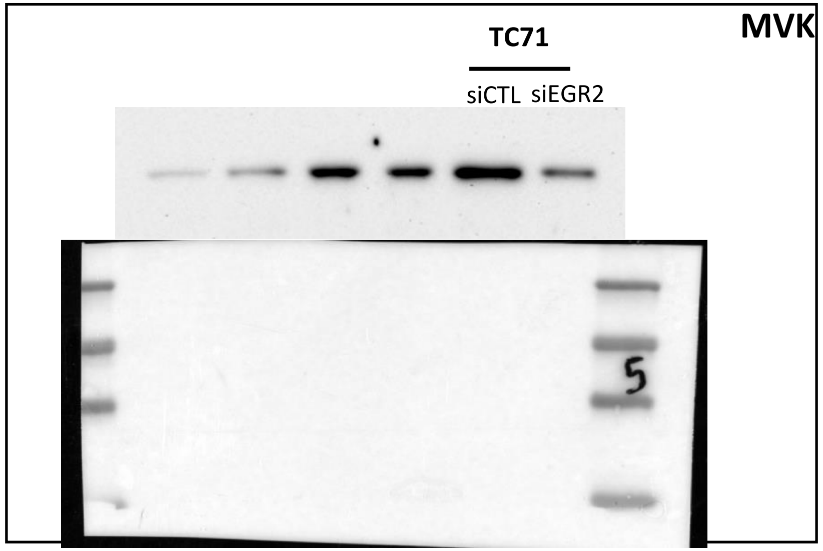

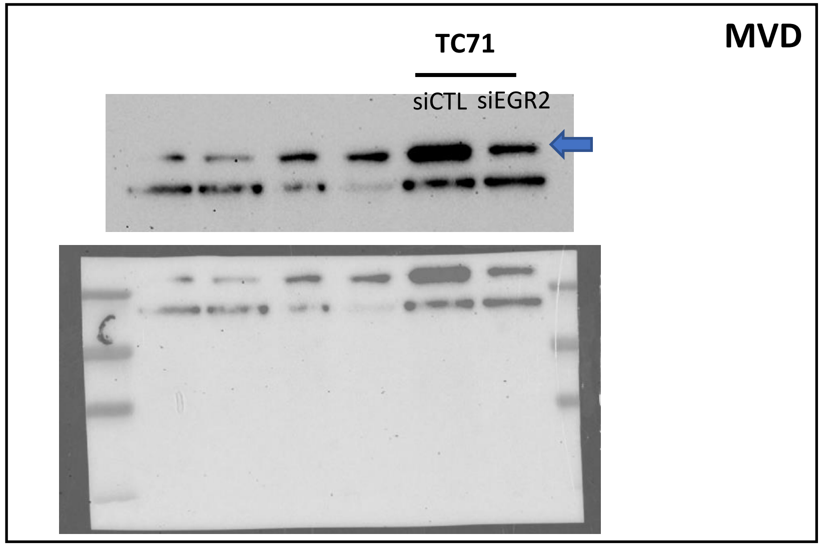

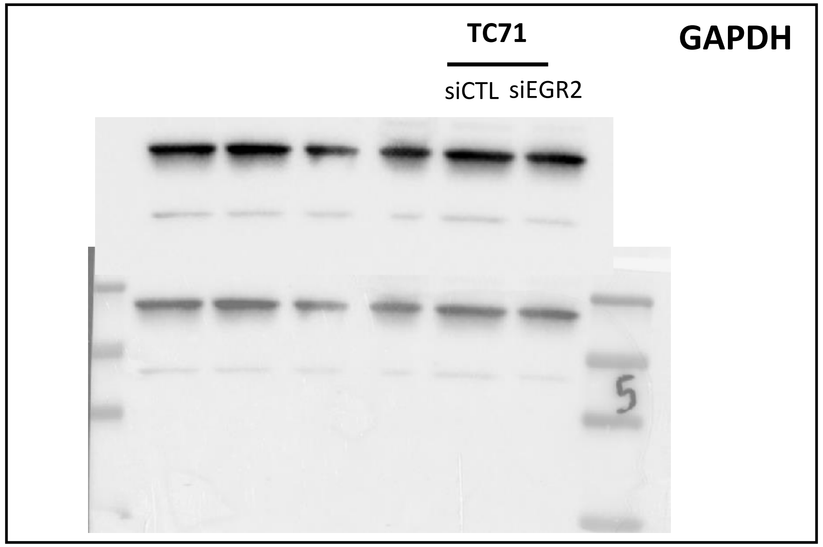

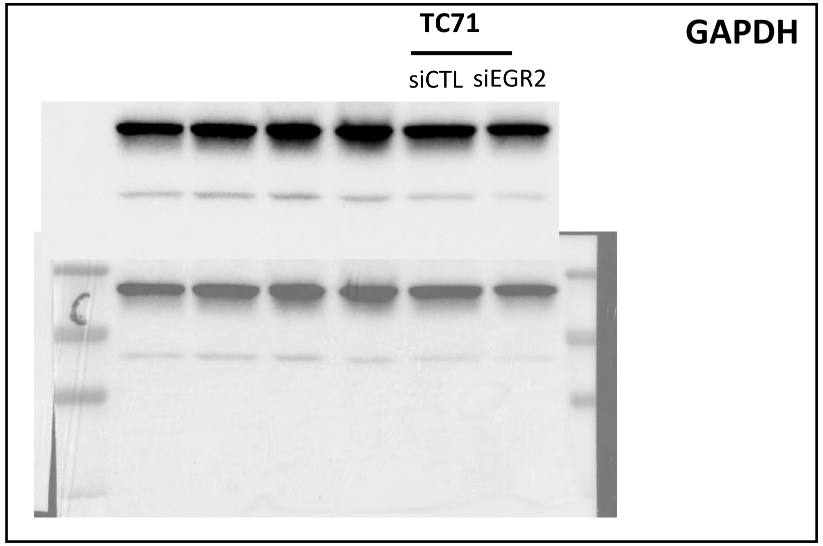

POE_siEGR2 figure 2B

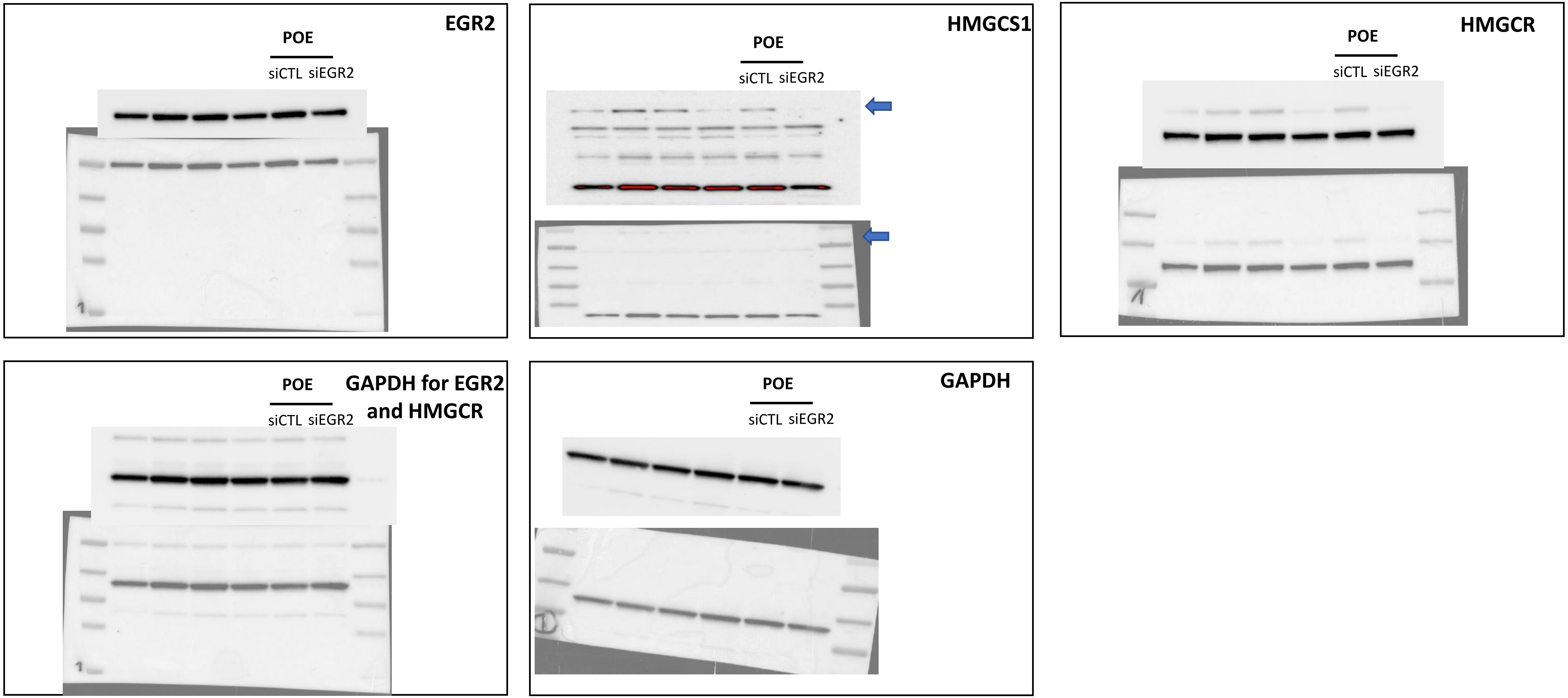

POE_siEGR2 figure 2B

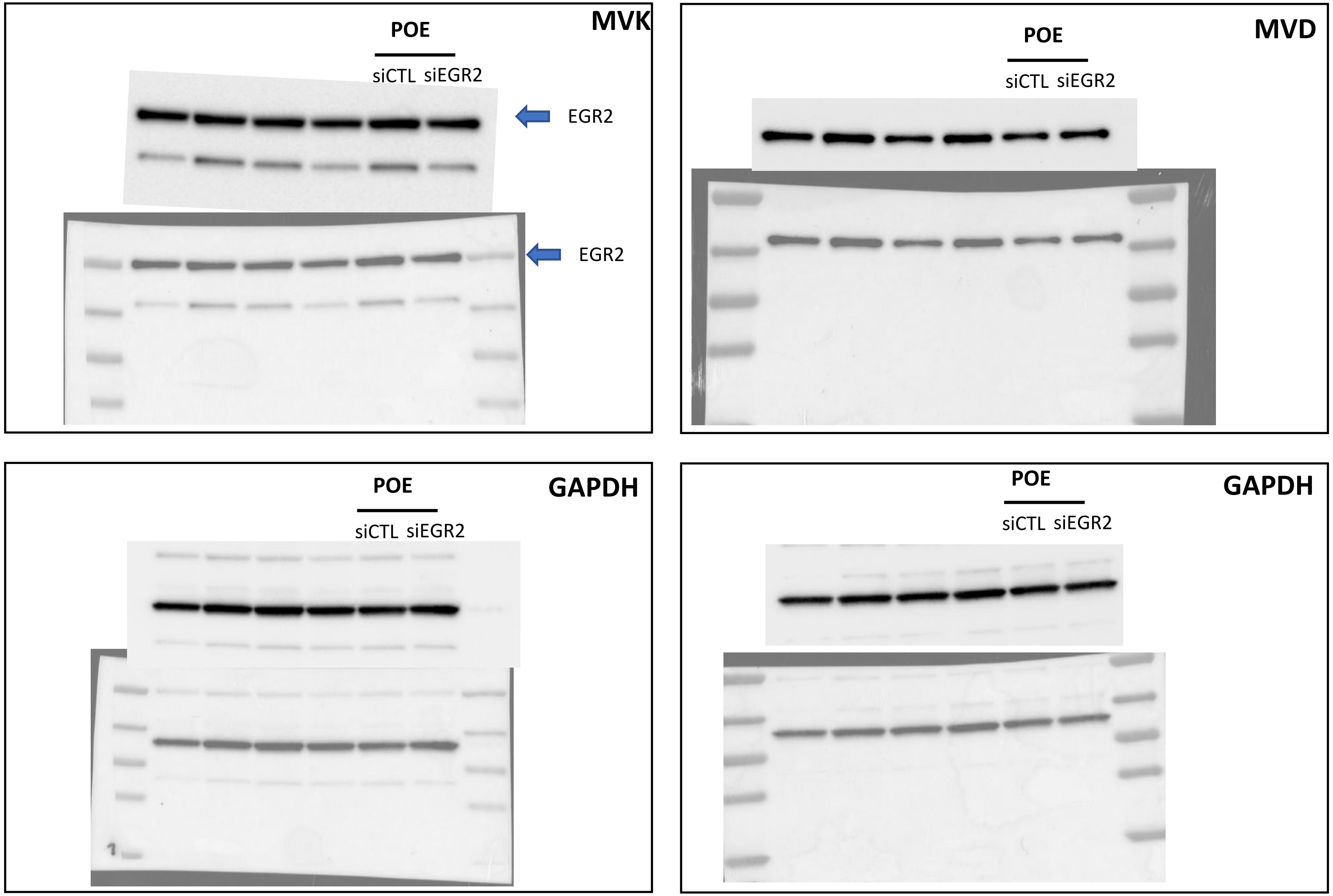

Figure 4F

Chemidoc acquisition

Chemiluminescence

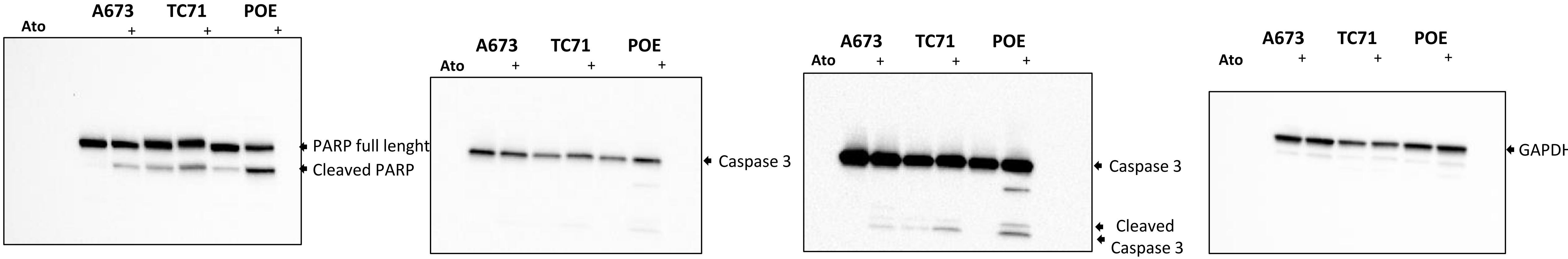

Composite

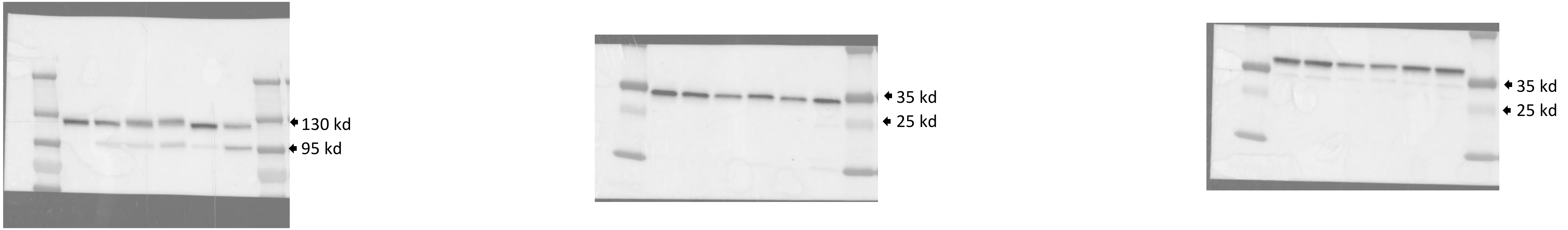

figure 5A

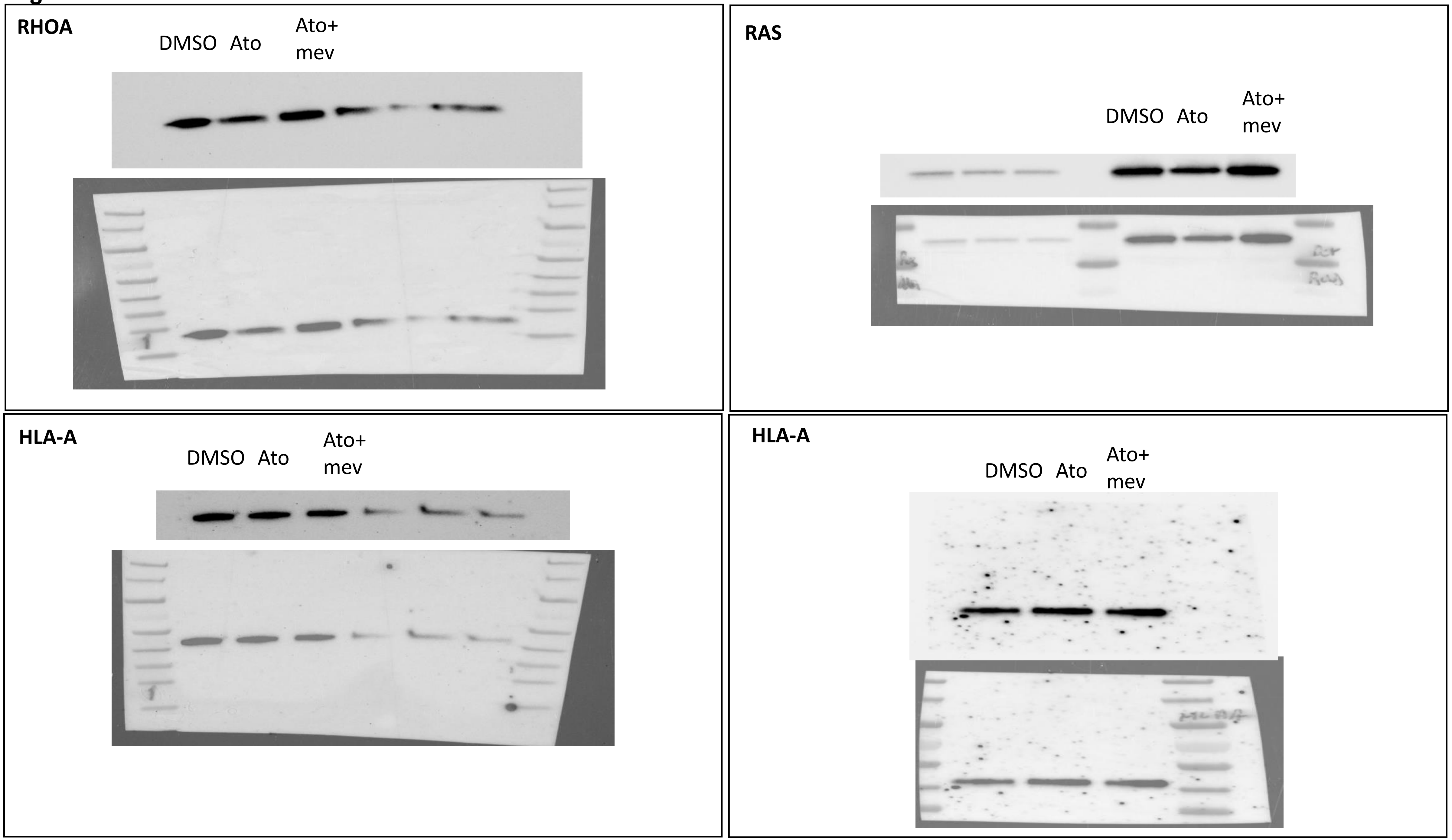
RHOA
DMSO
Ato
Ato+
mev
RAS
DMSO
Ato
Ato+
mev
HLA-A
DMSO
Ato
Ato+
mev
HLA-A
DMSO
Ato
Ato+
mev

figure 5A

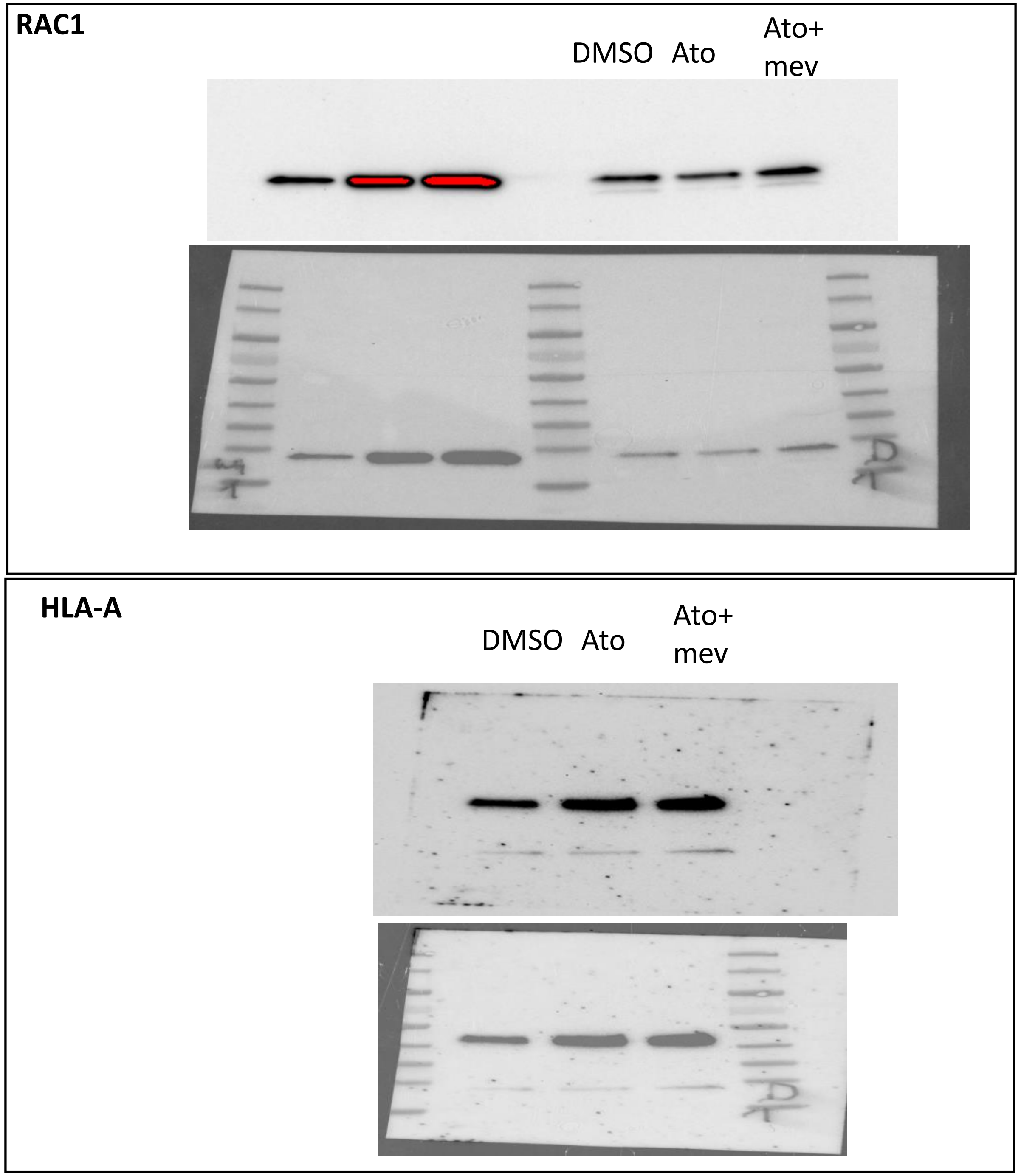

figure 5F and 5G

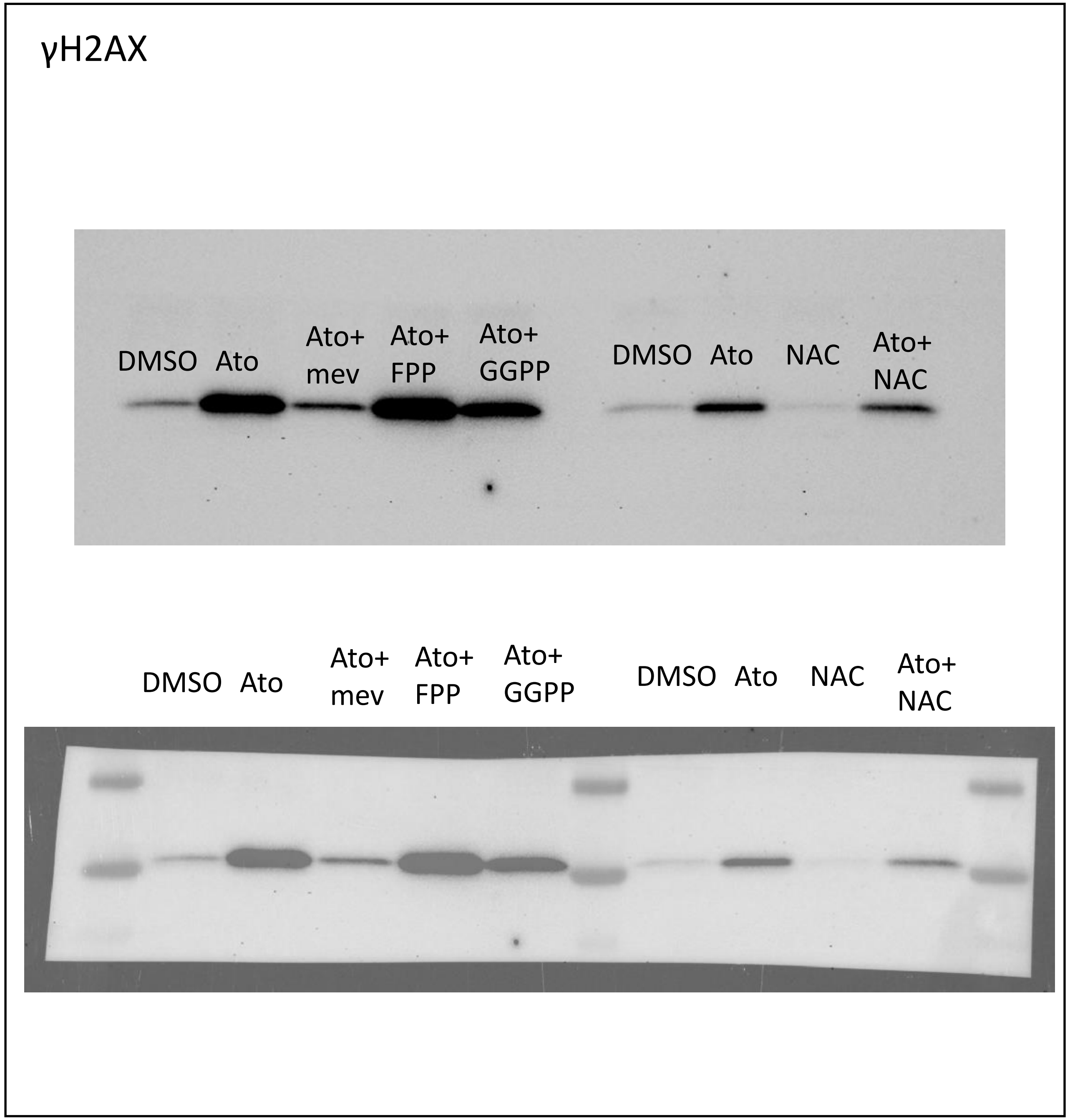

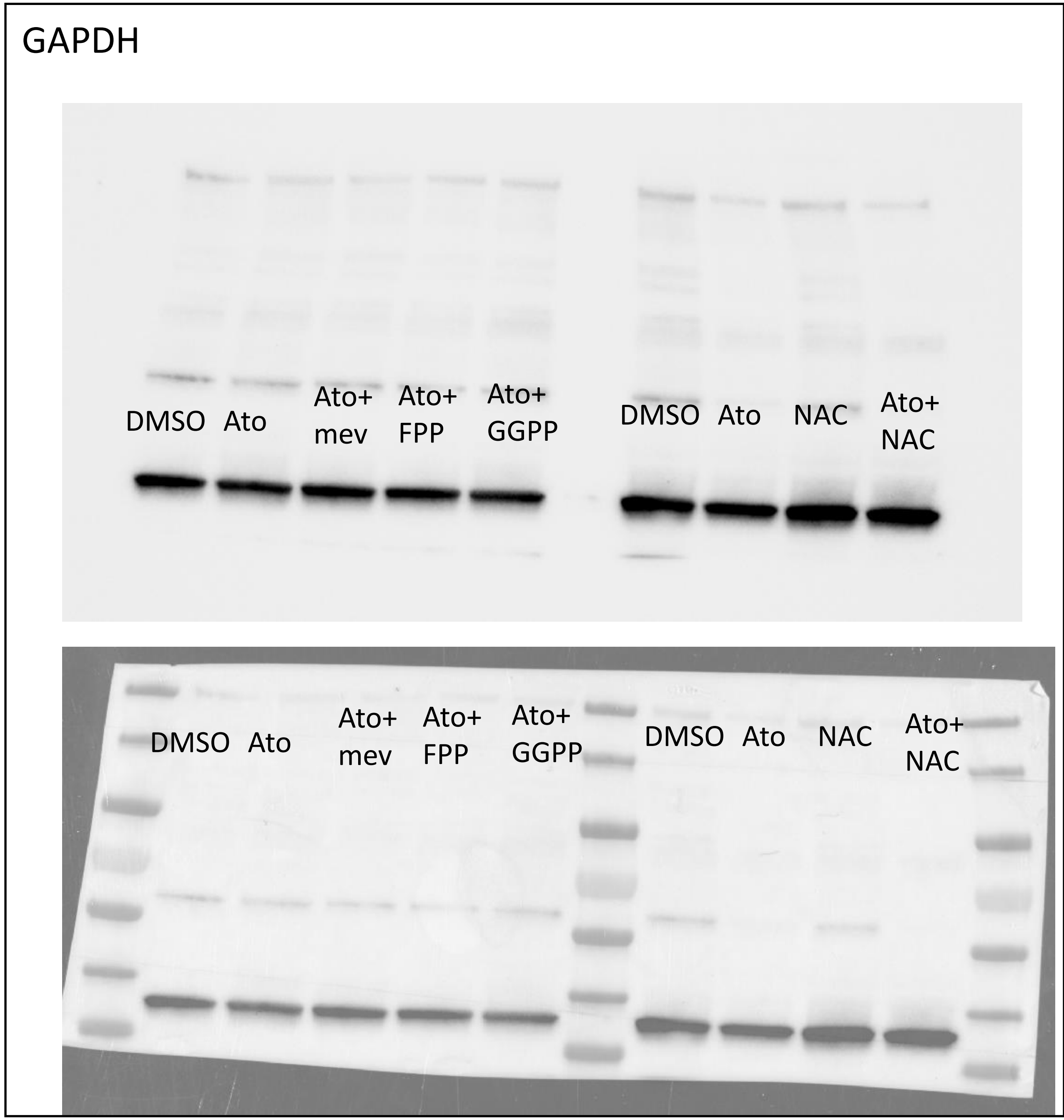

figure S1A

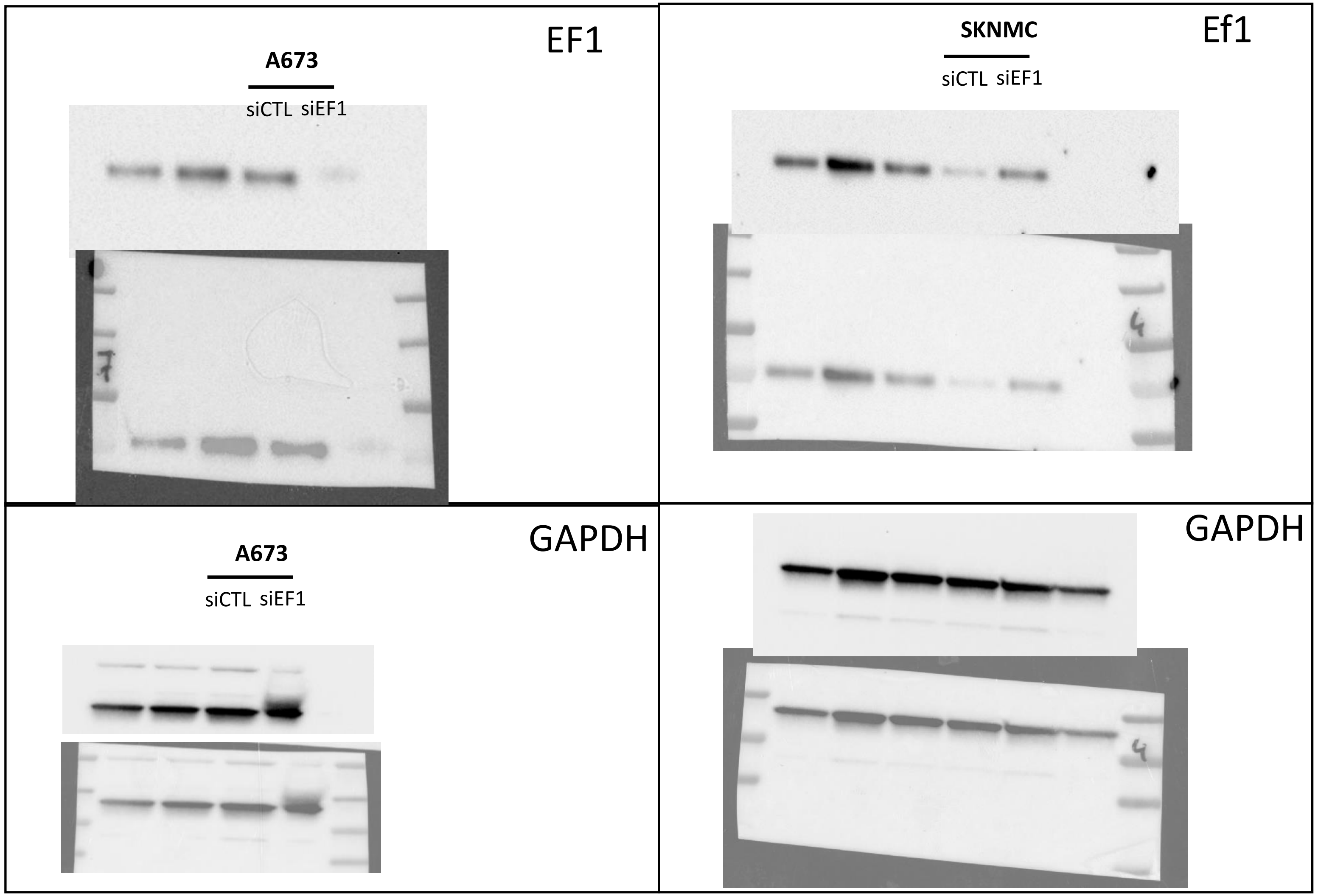
EF1
A673
siCTL siEF1
SKNMC
Ef1
siCTL siEF1
GAPDH
A673
siCTL siEF1
GAPDH
