# Supplementary figures and images for: Upregulation of the Mevalonate Pathway through EWSR1-FLI1/EGR2 Regulatory Axis Confers Ewing Cells Exquisite Sensitivity to Statins
Source: Cancers (Basel). 2022 May 8;14(9):2327. doi: 10.3390/cancers14092327 (PMC9100622; doi:10.3390/cancers14092327)

A

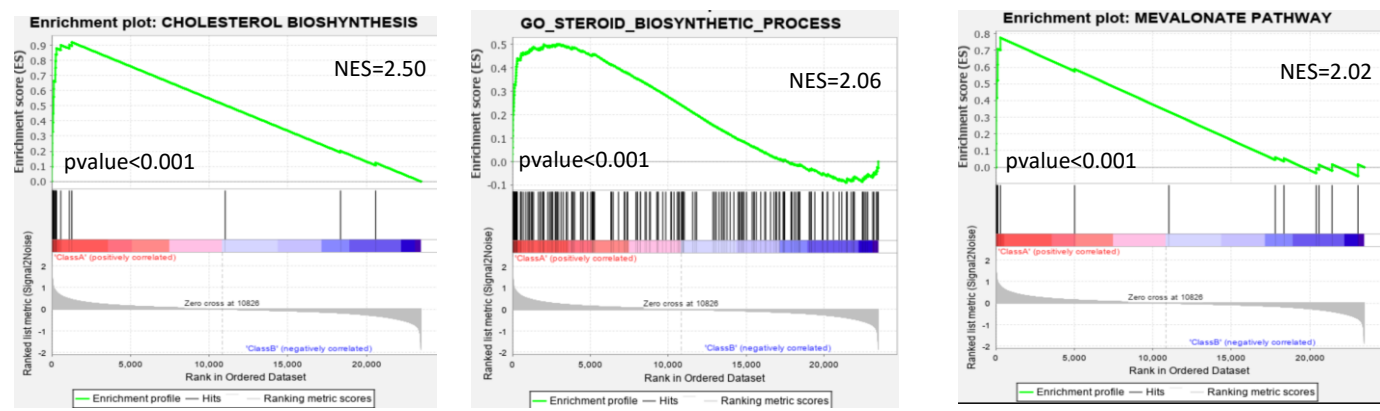

B

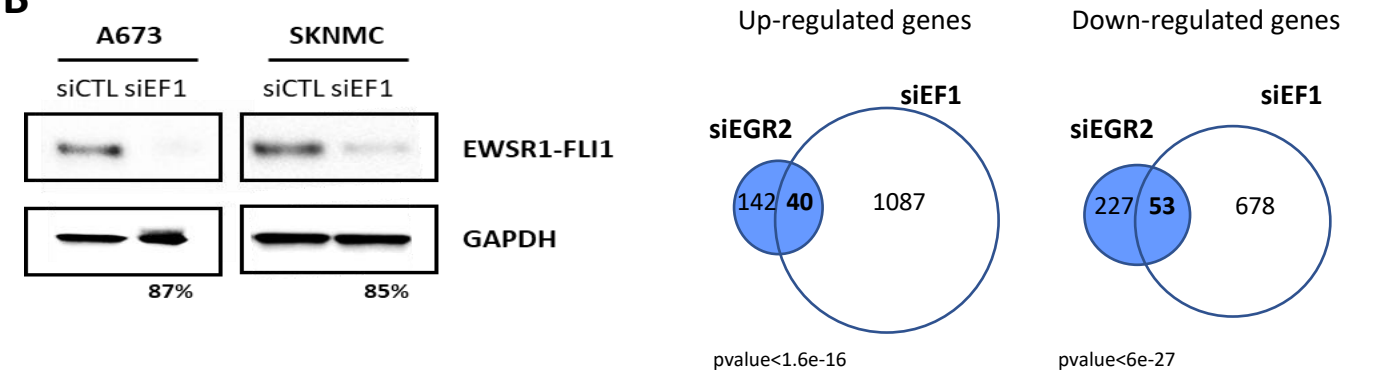

C

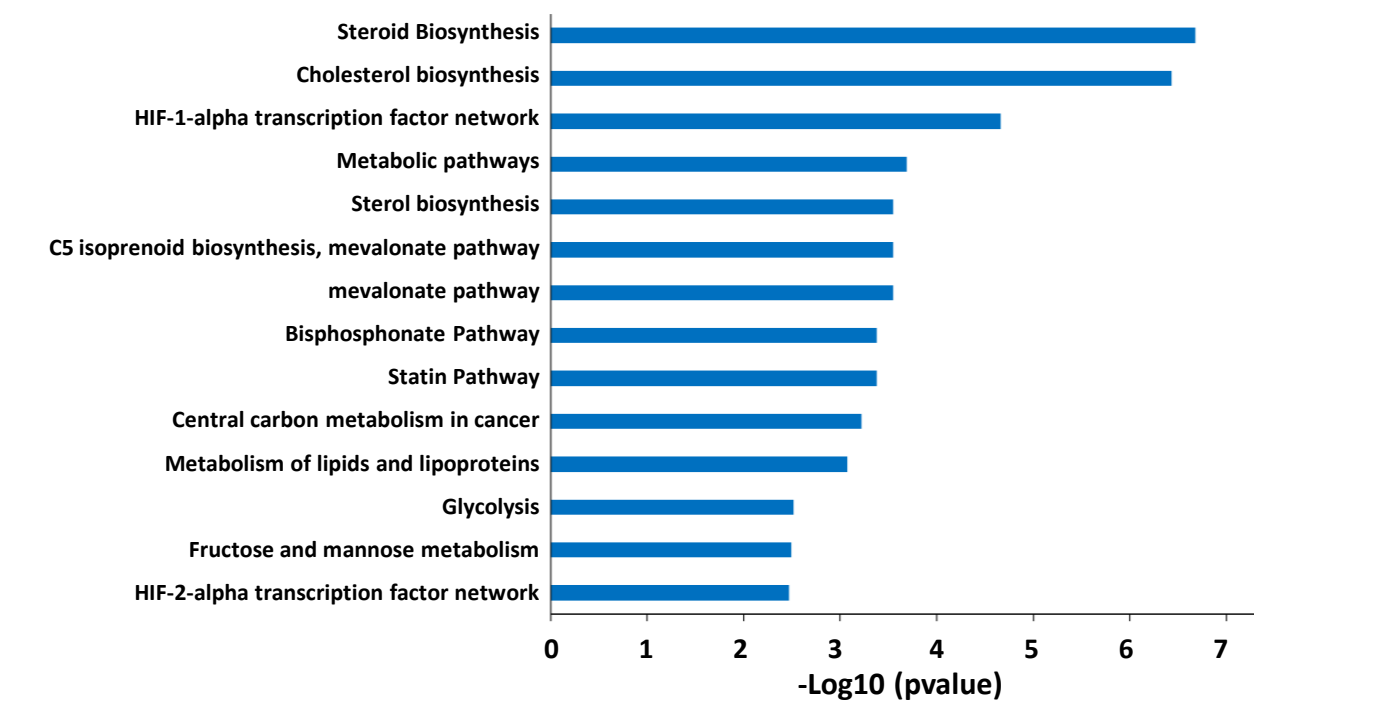

Supplement: Supplementary file 1 [file cancers-14-02327-s001.zip › Figure S1.pdf]

Supplementary Figure S2 related to Figure 3

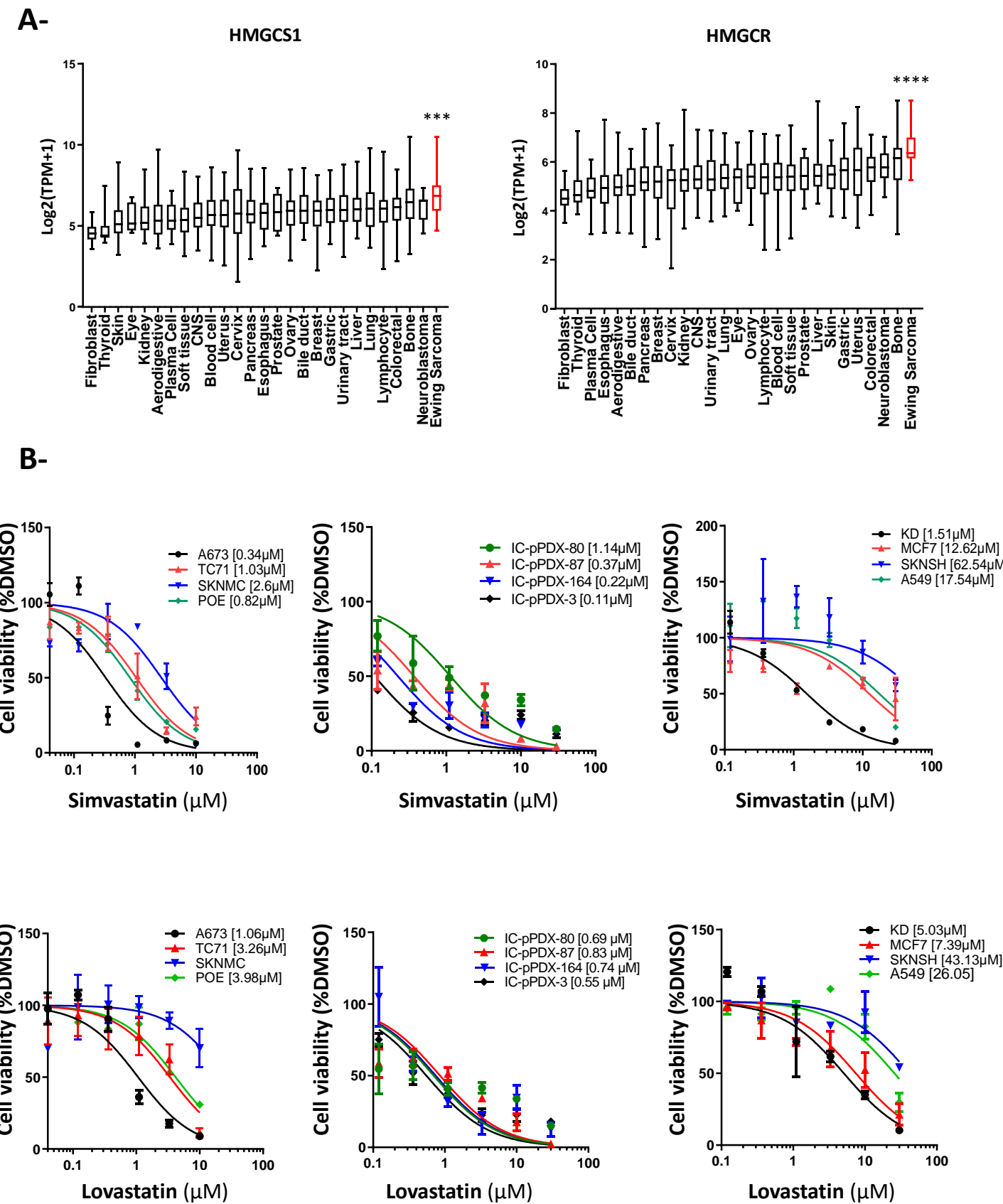

Supplement: Supplementary file 1 [file cancers-14-02327-s001.zip › Figure S2.pdf]

**A**

| RZ-score (24h) | Cell count | % of Growth |
|----------------|------------|-------------|
| 0              | 2472       | 100         |
| -2             | 2164       | 88          |
| -5             | 1771       | 72          |
| -10            | 1269       | 51          |
| -15            | 910        | 37          |

| RZ-score (72h) | Cell count | % of Growth |
|----------------|------------|-------------|
| 0              | 3102       | 100         |
| -2             | 2383       | 77          |
| -5             | 1626       | 52          |
| -10            | 886        | 29          |
| -15            | 500        | 16          |

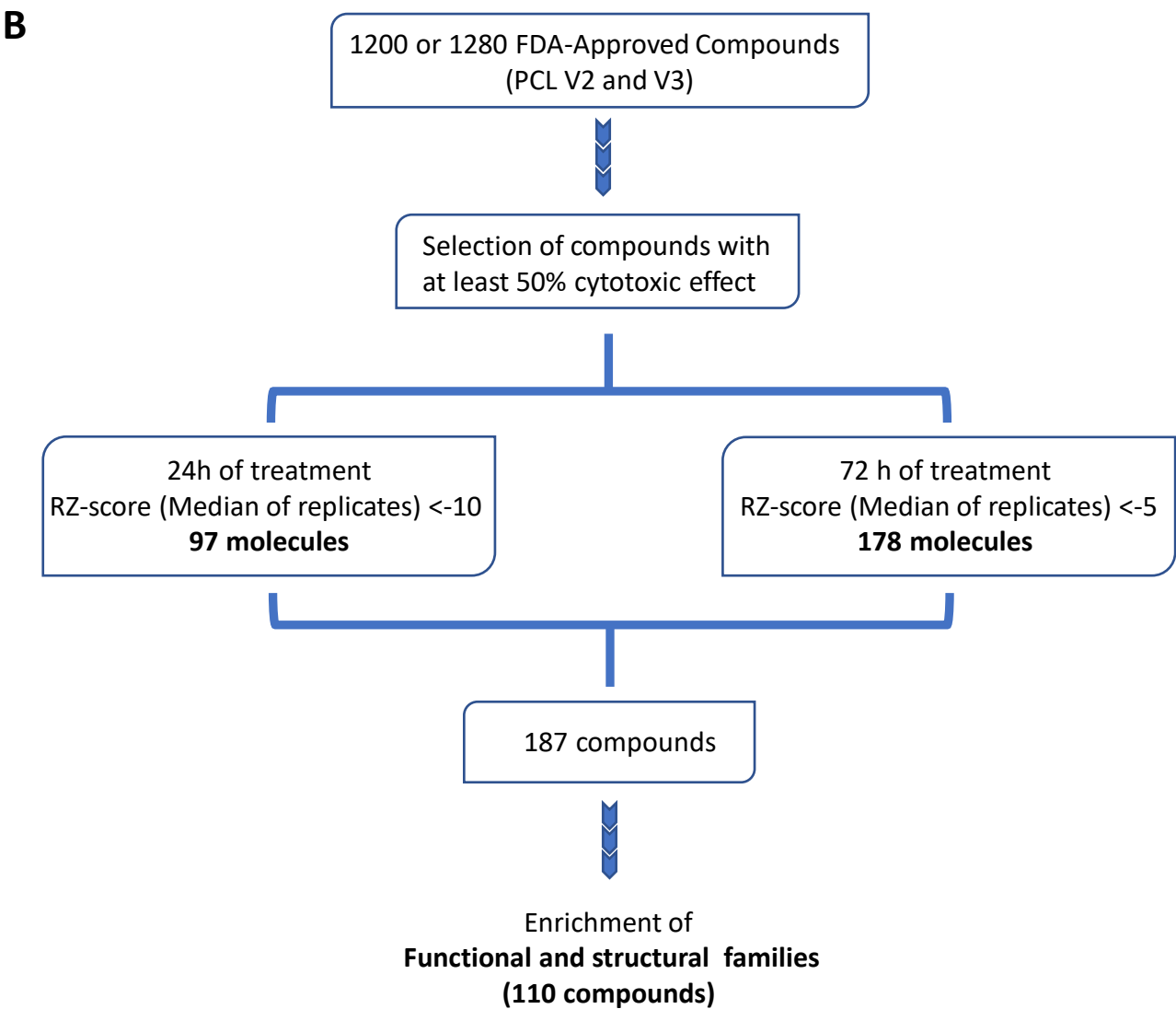

Supplement: Supplementary file 1 [file cancers-14-02327-s001.zip › Figure S3.pdf]

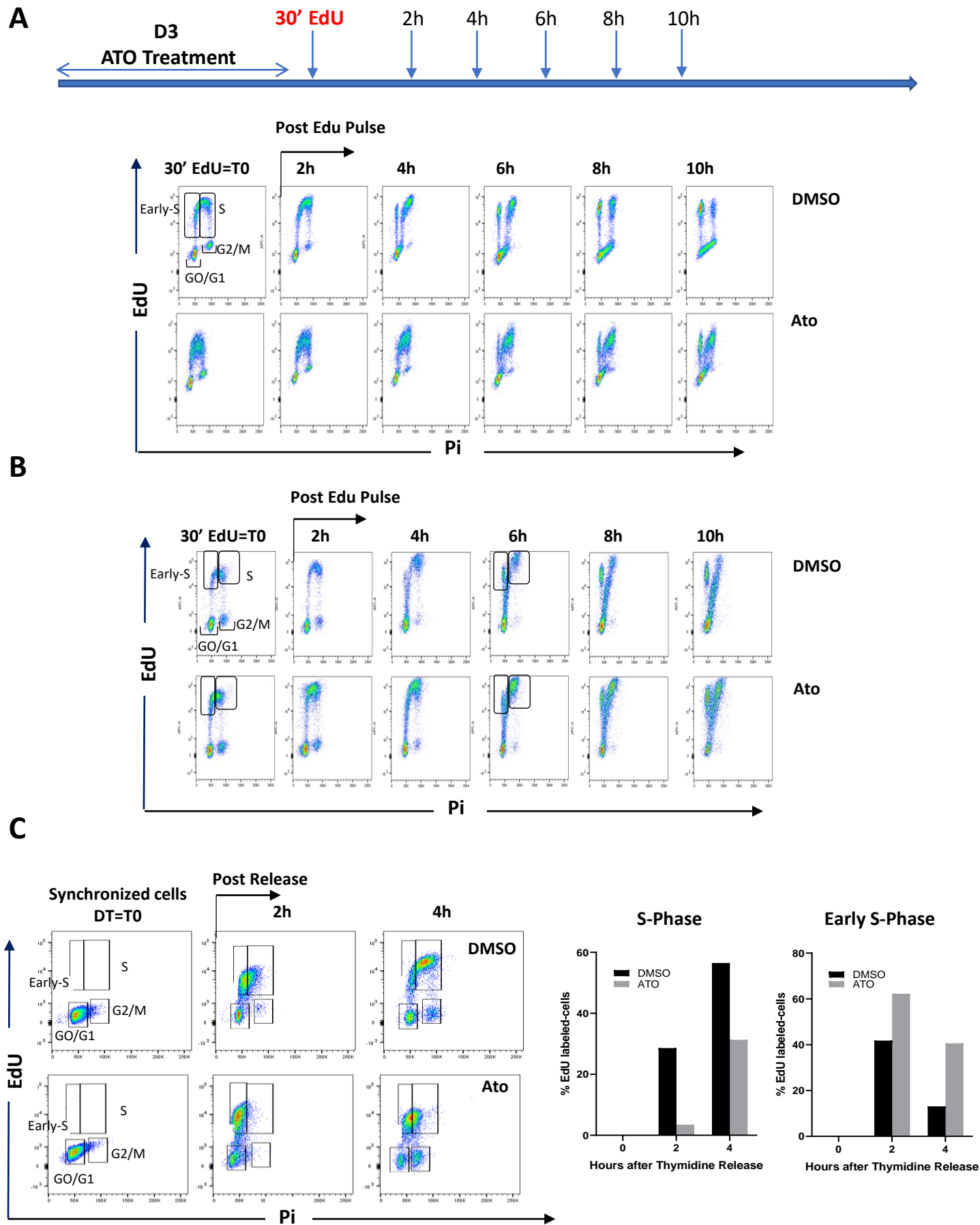

Supplement: Supplementary file 1 [file cancers-14-02327-s001.zip › Figure S4.pdf]
